# Extreme Diversity of Mycoviruses Present in Isolates of *Rhizoctonia solani* AG2-2 LP From *Zoysia japonica* From Brazil

**DOI:** 10.3389/fcimb.2019.00244

**Published:** 2019-07-12

**Authors:** Maria Aurea S. C. Picarelli, Marco Forgia, Eliana B. Rivas, Luca Nerva, Marco Chiapello, Massimo Turina, Addolorata Colariccio

**Affiliations:** ^1^Plant Virology Laboratory, Instituto Biológico, São Paulo, Brazil; ^2^Department of Life Science and System Biology, University of Turin, Turin, Italy; ^3^Institute for Sustainable Plant Protection, CNR, Turin, Italy; ^4^Phytopathological Diagnostic Laboratory, Instituto Biológico, São Paulo, Brazil; ^5^Council for Agricultural Research and Economics—Research Centre for Viticulture and Enology CREA-VE, Conegliano, Italy

**Keywords:** mycoviruses, *Rhizoctonia solani*, grass, multiple infection, viral diversity, phylogenetic analysis, virus taxonomy

## Abstract

*Zoysia japonica*, in Brazil, is commonly infected by *Rhizoctonia solani* (*R. solani*) in humid and cool weather conditions. Eight isolates of *R. solani*, previously identified as belonging to the AG2-2 LP anastomosis group, isolated from samples from large path symptoms, were collected from three counties in São Paulo state (Brazil) and investigated for the presence of mycoviruses. After detection of double-strand RNA (dsRNA) in all samples, RNA_Seq analysis of ribosomal RNA-depleted total RNA from *in vitro* cultivated mycelia was performed. Forty-seven partial or complete viral unique RNA dependent-RNA polymerase (RdRp) sequences were obtained with a high prevalence of positive sense ssRNA viruses. Sequences were sufficiently different from the first match in BLAST searches suggesting that they all qualify as possible new viral species, except for one sequence showing an almost complete match with Rhizoctonia solani dsRNA virus 2, an alphapartitivirus. Surprisingly four large contigs of putative viral RNA could not be assigned to any existing clade of viruses present in the databases, but no DNA was detected corresponding to these fragments confirming their viral replicative nature. This is the first report on the occurrence of mycoviruses in *R. solani* AG2-2 LP in South America.

## Introduction

*Zoysia japonica* (*Z. japonica*) Steud, especially from the cultivar “Esmeralda”, comprises 81% of the cultivated grasses in Brazil (Zanon, 2015). In 2015, the sod production of cultivated grasses reached 24,000 hectares (Antoniolli, 2015), which is an increase of 40% between 2010 and 2015 (Zanon, 2015). Nevertheless, it is very susceptible to the large patch disease caused by the fungus *Rhizoctonia solani* (*R. solani*) AG 2-2 LP, considered the most important disease of zoysia grass worldwide. Disease control is difficult, and practices employed in management are hardly effective, also, because fungicides are not approved on public areas, or home gardens. Considering these obstacles, biological control agents are desirable alternatives for disease management thanks to their environmental safety.

Since the first report in the 1960s, mycoviruses were searched for and found in many classes of phytopathogenic fungi, mostly because some mycoviruses can reduce the capacity of fungi to cause disease and may have the potential application as biological control agents. More recently, mycoviruses were also shown to be important for their environmental role and for modulating intra and inter-species interactions (Drinnenberg et al., 2011; Nerva et al., 2017, 2018a; Chun et al., 2018), possibly mediated by complex tripartite symbiotic relationships (Marquez et al., 2007). Not all the viruses associated with fungal pathogens affect virulence, but some can indeed cause hypovirulence, as is the case of the classic model system Cryphonectria hypovirus 1, infecting *Cryphonectria parasitica* (Nuss, 2005; Turina and Rostagno, 2007), or the association between Rhizoctonia solani partitivirus 2 (RsPV2) and *R. solani* AG-1 IA causing hypovirulence on rice (Zheng et al., 2014).

The presence of double-stranded RNAs (dsRNAs) is evidence of a mycovirus infection, which have been reported in many phytopathogenic fungi species and in different anastomosis groups from various hosts of *R. solani* (Strauss et al., 2000; Bharathan et al., 2005; Das et al., 2016; Zheng et al., 2018). More recently, ssDNA viruses were also shown to infect phytopathogenic fungi (Yu et al., 2010).

Nowadays, most approaches used to characterize fungal viruses rely on Next-Generation Sequencing (NGS) of total RNA depleted of ribosomal RNA or sequencing of small RNA (Vainio et al., 2015; Marzano and Domier, 2016; Marzano et al., 2016; Donaire and Ayllon, 2017). We directly compared the two methods in previous work and found that NGS of total RNA provides a more complete characterization of fungal associated viruses (Nerva et al., 2016).

Mycoviruses in single and mixed infections have been previously detected in *R. solani*: Rhizoctonia solani virus 717 (*Betapartitivirus*), Rhizoctonia solani RNA virus HN008, Rhizoctonia solani dsRNA virus 1, Rhizoctonia solani partitivirus 2, and Rhizoctonia solani flexivirus 1 (unclassified virus) (Zhong et al., 2015; Bartholomäus et al., 2016; Zheng et al., 2018). Moreover, recently, a plant virus—cucumber mosaic virus—was shown to accumulate and replicate in *R. solani* (Andika et al., 2017). Further NGS characterization of *R. solani*-associated viromes were performed on samples from USA (Marzano et al., 2016) and from samples from Germany (Bartholomäus et al., 2016).

In a preliminary screen, we detected the presence of dsRNAs in eight asymptomatic Brazilian *R. solani* AG2-2 LP isolates, a pathogen of *Z. japonica*, from three counties of São Paulo state (Picarelli, 2015). All the dsRNA electrophoretic patterns showed 3–6 bands with different sizes, all >2 kbp, and a fragment >8 kbp (Picarelli, 2015). These complex electrophoretic patterns could be due to the presence of segmented viral genomes, mixed infections, or defective dsRNAs. In this study, we aimed to characterize the virome associated with *R. solani*, isolated from *Z. japonica* grass that were positive in a preliminary dsRNA screen, to gather the first information about the diversity and the spread of mycoviruses in *R. solani* in different Brazilian regions. Although none of the isolates under scrutiny were hypovirulent, such a library of mycoviruses could be the basis for a targeted virus-induced gene silencing (VIGS) approach.

## Materials and Methods

### Fungal Isolates Origin and Growth Conditions

*Z. japonica* sheaths, showing large patch symptoms, were collected in three municipalities of São Paulo State, Brazil: Cotia (isolates IBRS07, IBRS15, IBRS16, and IBRS19), São Paulo (isolates IBRS04, IBRS22, and IBRS23), and Ilhabela (isolate IBRS11) (Supplementary Figure 1, online). All *R. solani* samples were collected from diseased patches of zoysia grass lawns showing the same characteristics. The eight *R. solani* AG2-2 LP isolates were maintained on potato dextrose agar medium at 25°C, for a 12 h photoperiod (Picarelli, 2015). Long-term conservation of the fungal isolates was obtained by growing the fungi on paper strips stored at −80°C or lyophilised mycelia after growth on potato dextrose broth and stored at −20°C.

### RNA Extraction, DNA Extraction, and cDNA Synthesis

Total RNA was extracted from 0.1 g of lyophilised fungal mycelium using the Spectrum™ Plant Total RNA Kit (Sigma-Aldrich, Darmstadt, Germany), according to the manufacturer's instructions. Copy DNA (cDNA) synthesis was performed using the High-Capacity cDNA Reverse Transcription Kit (Thermo Fisher Scientific, Waltham, MA, USA) as described in the kit's manual. DNA extraction was performed by breaking 50 mg of lyophilised mycelia in a bead beater, using 0.5 mm diameter glass beads, in a 2 mL Eppendorf tube with 700 μL of phenol and 700 μL of 2x STE-2%SDS. After centrifugation, the supernatant was collected and washed twice with chloroform-isoamyl alcohol, 24:1. The supernatant was then precipitated with 2 volumes of 100% ethanol and 0.1 volumes of 3 M sodium acetate, pH 5.2. The pellet was resuspended in 50 μL of H_2_O, quantified with a Nanodrop 2000 (Thermo Fisher Scientific, Waltham, USA) and diluted to 10 ng/μL for PCR applications.

### Library Preparations and Bioinformatic Analysis

Ribosomal RNA depletion, library preparations and Illumina sequencing were performed by Macrogen (Seoul, Republic of Korea); the assembly and virus identification steps were performed as previously described (Nerva et al., 2018b), where we have specified the details of commands and scripts used for each bioinformatics analysis step. Briefly, reads from RNA-Seq were assembled *de novo* using Trinity version 2.3.2 (Haas et al., 2013). Trinity assembly was then BLASTed against a custom viral database (https://osf.io/c9x2p/) to identify contigs of viral origin. The number of reads covering the viral genomes was obtained by mapping the reads from each sequenced library on reference sequences with Burrows-Wheeler Aligner (BWA) and Samtools (Li and Durbin, 2009; Li et al., 2009). The mapping step was performed as explained in detail previously (Nerva et al., 2018b) with only one exception: the bwa mem algorithm was used for the alignment instead of bwa aln. Mapping results were displayed using Tablet (Milne et al., 2013). Viral contigs displaying incomplete open reading frames (ORFs) and cut reads on the 5′ or 3′ ends were analyzed using MITObim (Hahn et al., 2013) to attempt extending the incomplete 5′ and 3′ ends. After one iteration, the eventually extended sequences were used as a query for a BLAST search against the trinity assembly to find contigs overlapping the extended region. Identified contigs were assembled using the CAP3 sequence assembly program (Huang and Madan, 1999).

### Quantitative RT-PCR Analysis

Primers for qRT-PCR were designed using Primer 3 (Untergasser et al., 2012), with the amplicon size between 70 and 120 bp. To associate specific RNA samples to each specific contig, qRT-PCR analysis were performed using a CFX Connect™ Real-Time PCR Detection System (Biorad, Hercules, USA). The PCR reaction was performed in 10 μL using the iTaq™ Universal SYBR® Green Supermix (Biorad, Hercules, USA). A melting curve analysis was performed at the end of the qRT-PCR protocol to check for unspecific PCR products. All the oligonucleotides used in the qRT-PCR protocol are reported in Supplementary Table 1, online.

### ORF Prediction and Phylogenetic Analyses

ORF predictions were performed using the ORF finder tool from NCBI, and predictions were made selecting the “standard” genetic code for all viral contigs, except the one closely related to mitoviruses, generally hosted in the mitochondria. These contigs were analyzed selecting the “Mold, protozoan and coelenterate mitochondrial” genetic code. The putative function of the predicted protein was established by BLAST analysis, looking at the function of the closest proteins in the NCBI database. The predicted protein sequences were analyzed through a BLASTP search using the domain finder option to evaluate the presence of any conserved domain in the sequence (such as the viral polymerase GDD conserved domain). Phylogenetic analyses were performed by aligning the viral RNA dependent-RNA polymerase (RdRp) proteins with MUSCLE implemented in MEGA6 (Tamura et al., 2013). Alignments were exported in FASTA format and submitted to the IQ-TREE web server (Trifinopoulos et al., 2016) to produce Maximum likelihood phylogenetic trees (Lam-Tung et al., 2015). The best substitution model was estimated automatically by IQ-TREE with ModelFinder (Kalyaanamoorthy et al., 2017) and ultrafast bootstrap analysis (Diep Thi et al., 2018) in which 1000 replicates were performed. For each tree, each specific model is indicated in the figure legend.

### Amplification and Cloning of Fragments From the Viral Genomes

To confirm the sequence of regions of interest in the assembled contigs, fragments from some of the viral genomes were amplified by designing PCR primers based on the *in silico* assembly and performing PCR reactions on cDNA produced as described above (Supplementary Table 2, online). Using electrophoresis, amplified bands were separated on an agarose gel. Cut bands were cleaned using Zymoclean Gel DNA Recovery Kit (Zymo Research, Irvine, USA), inserted in the pGEMT vector using pGEM®-T Easy Vector Systems (Promega, Madison, USA) and transformed in chemically competent *E. coli* DH5α (Mix & Go! E. coli Transformation kit, Zymo Research, Irvine, USA). Colonies that were positive for the insertion were selected for Sanger sequencing (Biofab S.r.l., Rome, Italy).

## Results and Discussion

A single sequencing run of the eight pooled *R. solani* isolates under scrutiny produced 167,355,298 total reads deposited in the SRA archive linked to BioProject PRJNA524447. After the Trinity run, a total of 89,779 contigs were assembled. A BLAST search of a custom prepared viral database identified a total of 56 putative viral contigs (Table 1). Among those, 44 contained the typical conserved motifs of a viral RdRp, which is essential for the replication of RNA viruses and often displays three conserved amino acids (GDD) that are crucial for the catalytic activity. Two are partial viral genomes, where the RdRp domain is probably located in the missing part, and one is a contig where the RdRp domain cannot be detected by search engines, but the protein sequence shows a similarity with the typical GDD RdRp domain from proteins belonging to the family *Hypoviridae*. Therefore, we identified at least 47 distinct viruses (Table 1). There were five contigs encoding Coat Protein (CP) of bipartite viruses, and finally four putative viral contigs, encoding for proteins of unknown function (ORFans). We then checked the association of each contig with each of the eight isolates using qRT-PCR specific for each fragment; the results are displayed in Table 2. The number of virus contigs/isolate varies from isolate IBRS15 containing 6 viral contigs to isolate IBRS23 containing 30 viral contigs.

**Table 1:** List of viruses discovered in *Rhizoctonia solani* isolates from São Paulo State (Brazil).

**Virus name**		**NCBI ID**	**Abbreviation**	**Segment length (bp)**	**No. of reads**	**BLASTx first hit**	**Ident**	**Query cover**
Rhizoctonia solani endornavirus 4		MK393902	RsEV4	20,215	23,587	Endornavirus-like virus	35.16	23
Rhizoctonia solani endornavirus 5		MK393903	RsEV5	16,227	6,414	Rhizoctonia cerealis alphaendornavirus 1	47.31	36
Rhizoctonia solani endornavirus 6		MK393904	RsEV6	15,273	4,441	Morchella importuna endornavirus 2	45.18	49
Rhizoctonia solani endornavirus 7		MK393905	RsEV7	14,325	73,559	Rhizoctonia solani endornavirus 2	30.69	83
Rhizoctonia solani partitivirus 8	RNA1	MK532273	RsPV8	1,958	2,239	Rhizoctonia oryzae-sativae partitivirus 1	53.19	90
	RNA2	MK532274		778	178	Heterobasidion partitivirus 20	42.35	74
Rhizoctonia solani partitivirus 6	RNA1	MK507781	RsPV6	2,401	5,189	Fusarium poae partitivirus 2	50.96	90
	RNA2	MK507782		2,310	11,867	Rosellinia necatrix partitivirus 8	58.08	84
Rhizoctonia solani partitivirus 7	RNA1	MK507783	RsPV7	1,912	10,488	Trichoderma atroviride partitivirus 1	68.08	95
	RNA2	MK507784		1,867	3,218	Trichoderma atroviride partitivirus 1	31.69	86
Rhizoctonia solani dsRNA virus 2	RNA1	MK400668	RsdsRNA2	1,942	32,967	Rhizoctonia solani dsRNA virus 2	99.36	96
	RNA2	MK400669		1,727	16,114	Rhizoctonia solani dsRNA virus 2	98.77	84
Rhizoctonia solani bipartite-like virus 1	RNA1	MK492913	RsBLV1	1,827	279	Ceratobasidium mycovirus-like	71.50	69
	RNA2	MK492914		1,888	331	Ceratobasidium mycovirus-like	61.60	41
Rhizoctonia solani dsRNA virus 6		MK507788	RsdsRNA6	11,847	5,257	Rhizoctonia fumigata mycovirus	30.95	20
Rhizoctonia solani dsRNA virus 7		MK507789	RsdsRNA7	3,523	1,839	Rhizoctonia fumigata mycovirus	31.70	78
Rhizoctonia solani dsRNA virus 8		MK507790	RsdsRNA8	2,905	1,370	Rhizoctonia fumigata mycovirus	30.83	85
Rhizoctonia solani dsRNA virus 9		MK507791	RsdsRNA9	2,162	445	Rhizoctonia fumigata mycovirus	35.48	70
Rhizoctonia solani dsRNA virus 10		MK532272	RsdsRNA10	9,416	3,945	Sclerotium rolfsii mycovirus dsRNA 1	37.37	37
Rhizoctonia solani beny-like virus 1		MK507778	RsBeLV1	11,666	11,135	Sclerotium rolfsii beny-like virus 1	36.35	39
Rhizoctonia solani bunya/phlebo-like virus 1		MK507779	RsBPLV1	7,804	25,865	Barns Ness serrated wrack bunya/phlebo-like virus 1	30.56	60
Rhizoctonia solani flexi-like virus 1		MK507787	RsFLV1	2,982	1,848	Rhizoctonia solani flexivirus 2	70.50	27
Rhizoctonia solani alphavirus-like 1		MK507793	RsALV1	2,414	949	Rhizoctonia solani RNA virus 3	82.14	38
Rhizoctonia solani alphavirus-like 2		MK507792	RsALV2	3,396	509	Rhizoctonia solani RNA virus 1	69.38	27
Rhizoctonia solani alphavirus-like 3		MK507786	RsALV3	6,752	924	Rhizoctonia solani RNA virus 3	83.12	13
Rhizoctonia solani mitovirus 21		MK372892	RsMV21	4,100	20,0,8721	Rhizoctonia solani mitovirus 13	51.13	66
Rhizoctonia solani mitovirus 22		MK490928	RsMV22	2,177	27,09274	dsRNA viral RdRp (mitochondrion) [Thanatephorus cucumeris]	80.15	73
Rhizoctonia solani mitovirus 23		MK375261	RsMV23	2,792	16,946	Ceratobasidium mitovirus A	61.98	83
Rhizoctonia solani mitovirus 24		MK372893	RsMV24	2,149	7,52995	Macrophomina phaseolina mitovirus 2	85.48	75
Rhizoctonia solani mitovirus 25		MK372894	RsMV25	3,767	24,180	Macrophomina phaseolina mitovirus 3	38.49	43
Rhizoctonia solani mitovirus 26		MK372895	RsMV26	2,580	94,168	Rhizoctonia cerealis mitovirus	46.15	90
Rhizoctonia solani mitovirus 27		MK372896	RsMV27	3,176	11,379	Rhizoctonia cerealis mitovirus	50.42	77
Rhizoctonia solani mitovirus 28		MK372897	RsMV28	2,640	2,693	Rhizoctonia mitovirus 1	39.85	76
Rhizoctonia solani mitovirus 29		MK372898	RsMV29	2,904	56,286	Binucleate Rhizoctonia mitovirus K1	42.14	79
Rhizoctonia solani mitovirus 30		MK372899	RsMV30	2,760	3,461	Binucleate Rhizoctonia mitovirus K1	42.74	80
Rhizoctonia solani mitovirus 31		MK372900	RsMV31	3,820	16,05182	Rhizoctonia solani mitovirus 7	52.80	69
Rhizoctonia solani mitovirus 32		MK372901	RsMV32	3,409	14,32655	Rhizoctonia solani mitovirus 7	42.81	73
Rhizoctonia solani mitovirus 33		MK372902	RsMV33	2,733	50,5184	Rhizoctonia solani mitovirus 7	37.97	69
Rhizoctonia solani mitovirus 34		MK372903	RsMV34	3,389	10,356	Rhizoctonia solani mitovirus 11	44.00	71
Rhizoctonia solani mitovirus 35		MK490929	RsMV35	3,772	24,59926	Rhizoctonia solani mitovirus 13	43.70	67
Rhizoctonia solani mitovirus 36		MK490930	RsMV36	2,562	9,58130	Rhizoctonia solani mitovirus 13	54.90	71
Rhizoctonia solani mitovirus 37		MK372904	RsMV37	3,597	3,79328	Rhizoctonia solani mitovirus 15	51.06	69
Rhizoctonia solani mitovirus 38		MK372905	RsMV38	3,197	24,67420	dsRNA viral RdRp (mitochondrion) [Thanatephorus cucumeris]	45.42	66
Rhizoctonia solani ourmia-like virus 2		MK372906	RsOLV2	4,104	22,042	Rhizoctonia solani ourmia-like virus 1 RNA 1	78.18	44
Rhizoctonia solani ourmia-like virus 3		MK372907	RsOLV3	3,223	3,795	Rhizoctonia solani ourmia-like virus 1 RNA 1	76.59	57
Rhizoctonia solani ourmia-like virus 4		MK372909	RsOLV4	4,557	5,471	Rhizoctonia solani ourmia-like virus 1 RNA 1	48.96	34
Rhizoctonia solani ourmia-like virus 5		MK372908	RsOLV5	5,234	3,868	Agaricus bisporus virus 15	25.89	29
Rhizoctonia solani fusarivirus 1		MK558257	RsFV1	10,776	2,788	Rosellinia necatrix fusarivirus 2	40.00	25
Rhizoctonia solani fusarivirus 2		MK558256	RsFV2	10,710	36,543	Rosellinia necatrix fusarivirus 2	40.57	25
Rhizoctonia solani fusarivirus 3		MK558258	RsFV3	5,959	8,858	Fusarium graminearum dsRNA mycovirus-1	38.09	57
Rhizoctonia solani hypovirus 1		MK558259	RsHV1	18,371	86,930	Sclerotium rolfsii hypovirus 1	27.35	32
Rhizoctonia solani hypovirus 2		MK558260	RsHV2	9,606	4,582	Sclerotium rolfsii hypovirus 1	27.16	24
Rhizoctonia solani hypovirus 3		MK558255	RsHV3	5,518	1,553	Agaricus bisporus virus 2	28.49	18
Rhizoctonia solani putative virus 1		MK507780	RsPuV1	6,311	56,781	Lily symptomless virus	24.91	9
Rhizoctonia solani putative virus 2		MK507785	RsPuV2	7,137	18,568	Sanxia atyid shrimp virus 1	22.73	9
Rhizoctonia solani putative virus 3		MK532275	RsPuV3	7,214	3,662	Guarapuava tymovirus-like 1	31.91	9
Rhizoctonia solani putative virus 4		MK507793	RsPuV4	7,833	7,495	Gayfeather mild mottle virus	25.28	9

*For each virus the NCBI code is reported together with the abbreviation used in the paper, segment length, number of reads mapping the segment, and first hit obtained from BLASTx analysis with query cover and identity percentages*.

**Table 2:** List of Ct (threshold cycles) obtained from qRT-PCR analysis for each viral genome segment in every fungal isolate investigated.

**Viral contig**		***Rhizoctonia solani*** **isolates**							
		**IBRS22**	**IBRS23**	**IBRS04**	**IBRS07**	**IBRS11**	**IBRS15**	**IBRS16**	**IBRS19**
RsEV4					27			26	28
RsEV5		28	31	25	28			30	
RsEV6									27.8
RsEV7		25	24	22	24	24	24	23	24
RsPV6	RNA1		22						
	RNA2		19.4						
RsPV7	RNA1		24						
			27						
RsPV8	RNA1							23	
	RNA2							27	
RsdsRNA2	RNA1	24	29	22	27			29	24
	RNA2	29	30	21	26			25	23
RsBLV1	RNA1					28			
	RNA2					28.5			
RsdsRNA6		31	27	27.5	27.5			33	
RsdsRNA7			30	32	30			30	
RsdsRNA8			31	32	33			34	
RsdsRNA9			29	32				30	
RsdsRNA10		30	29		30	29		29	28
RsBeLV1									22
RsBPLV1			20.48						
RsFLV1					30				31
RsALV1									28.5
RsALV2			30			30			30
RsALV3			29						
RsMV21			15.5						
RsMV22			18		18	16			30
RsMV23					14.5				
RsMV24		26	24	29	25	23	25.5	26	24
RsMV25				26					
RsMV26				25					
RsMV27				27					
RsMV28									21
RsMV29		24.5	22.5	28	22	21	21.5	22.5	22.5
RsMV30									21.3
RsMV31					14				
RsMV32			15						
RsMV33				25					
RsMV34				29					
RsMV35				27	29				17
RsMV36			18						
RsMV37			22	28	23	23			
RsMV38			17		16		34		
RsOLV2			20			16		26	21
RsOLV3					24				28
RsOLV4		30.5	25		25			30.5	27
RsOLV5					24.44				
RsFV1							26		
RsFV2		26.5	25		25.5	24		26	26.5
RsFV3			29	29	29				28
RsHV1								33	19
RsHV2								23	
RsHV3								24.55	
RsPuV1		23	22	28	21.5		21	22	
RsPuV2								24	20
RsPuV3		29	26	34	25.5			26.5	26
RsPuV4					22				

*Empty boxes have been left where no specific amplification curve was detected*.

A quantitative estimation of the abundance of each contig can also be inferred by the number of mapped reads on each segment (Table 1).

The retrieved contigs showing homology with RdRps fell in 6 from the 16 clades that accommodate the overall viral diversity of RNA viruses of invertebrates (Shi et al., 2016).

### Narna-Levi Related Sequences

**Figure 1:**
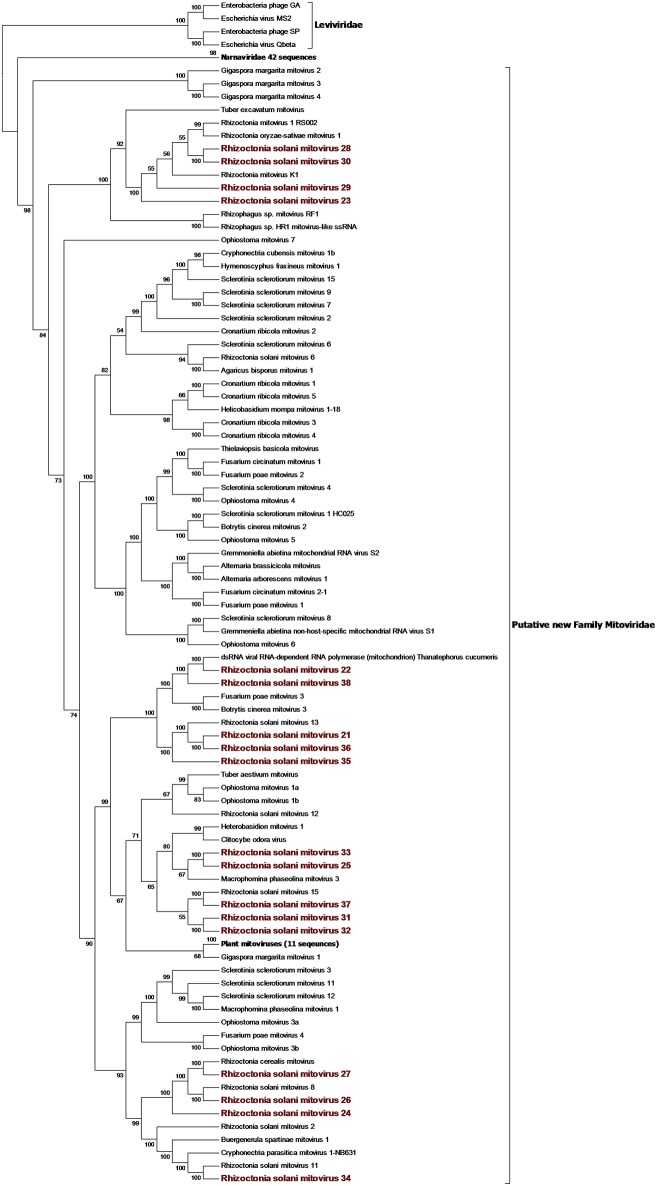
Phylogenetic analysis of positive sense RNA viruses related to the genus *Mitovirus*. 142 sequences have been used to produce an alignment starting from viruses belonging to the family *Narnaviridae, Botourmiaviridae*, and *Leviviridae* as outgroup; the phylogenetic tree was built using the maximum likelihood method, the best choice for the substitution model according to ModelFinder was PMB+F+I+G4. Ultrafast bootstrap analysis was performed with 1,000 replicates, and branches displaying values below 50 were collapsed. Viruses discovered in this work are in bold red ink; 42 sequences belonging to the *Narnaviridae* family have been compressed in one branch. A list of accession numbers of the sequences used for this analysis can be found in Supplementary Table 3, online. Detailed information about softwares used for the analysis can be found in the material and methods section.

**Figure 2:**
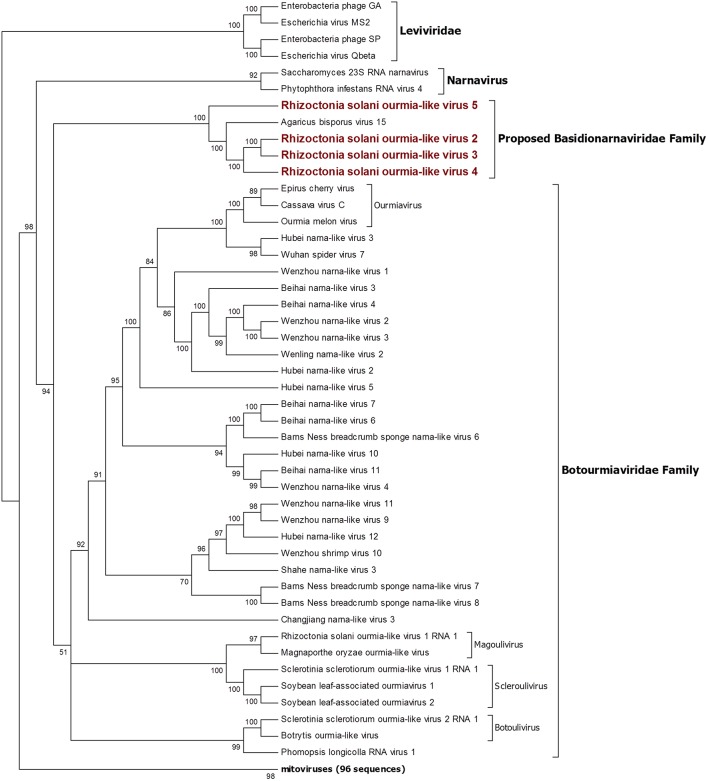
Phylogenetic analysis of positive sense RNA viruses related to the genus *Botourmiaviridae*. 142 sequences have been used to produce an alignment starting from viruses belonging to the family *Narnaviridae, Botourmiaviridae*, and *Leviviridae* as outgroup; phylogenetic tree was built using the maximum likelihood method the best choice for the substitution model according to ModelFinder was PMB+F+I+G4. Ultrafast bootstrap analysis was performed with 1,000 replicates, and branches displaying values below 50 were collapsed. Viruses discovered in this work are in bold red ink; 96 sequences belonging to the genus *Mitovirus* have been compressed in one branch. A list of accession numbers of the sequences used for this analysis can be found in Supplementary Table 3, online. Detailed information about softwares used for the analysis can be found in the material and methods section.

From the RNAseq assembly, we identified 22 sequences encoding for proteins showing high similarity with viruses from the Narna-levi clade (Shi et al., 2016). All these sequences encode for one ORF producing the putative RdRp; however, at least four of them were not complete. Since the RdRp domain was still detectable in the partial sequences, we included them in our phylogenetic analysis (Figures 1, 2). The predicted protein alignment and phylogenetic analysis showed that 18 sequences appeared to be part of the genus *Mitovirus*, while the 4 remaining were strongly related to ourmia-like viruses, but not included in the currently recognized *Narnavirus* genus. Thus, sequences were named as Rhizoctonia solani mitovirus 21 to 38 and Rhizoctonia solani ourmia-like virus 2 to 5, since previous work had already identified Rhizoctonia solani mitoviruses and ourmia-like viruses (Lakshman et al., 1998; Bartholomäus et al., 2016; Marzano et al., 2016). The amino acid sequence identity against the first hit in a BLAST search ranged from 80% (in the case of RsMV22 and a dsRNA viral element discovered in the same species) to 25.89% (in the case of RsOLV5 and Agaricus bisporus virus 15). From the phylogenetic tree (Figure 1), it is possible to observe that the mitoviruses detected in *R. solani* gather in four distinct sub-clades. Furthermore, we confirmed the necessity to revise the overall taxonomy of Levi-Narna viruses: the putative order Narnavirales should be established with a number of families, including the *Narnaviridae* (with the current *Narnavirus* genus) and the proposed/putative Mitoviridae (the current *Mitovirus* genus). The proposed new family Mitoviridae should be subdivided into a number of genera, including plant and fungal mitoviruses (Nibert et al., 2018; Nerva et al., 2019). The fact that most *R. solani* mitoviruses fall in the same three clades is probably due to the fact that mitoviruses are located and replicate in the host mitochondria and interspecific transmission does not easily occur in nature. Nevertheless, at least four clades mixed with viruses from ascomycetes and basidiomycetes occur, suggesting that some horizontal transfer can still occur. Further pairwise comparison among the distinct viral contigs from the Trinity assembly showed that RsMV 21 and 36 and RsMV 28 and 30 were almost completely identical (98 and 100% at the nucleotide (nt) level, respectively, for the two pairs in the conserved regions), but RsMV28 had a 120-nt deletion in position 504 of the genome. Rhizoctonia solani mitovirus 22 encodes for an RdRp showing 80% identity with the ORF characterized from a dsRNA element isolated from a hypovirulent strain of *R. solani* in 1998 (Lakshman et al., 1998). Since this sequence is still annotated in the NCBI database, as a viral dsRNA element located in the fungal mitochondria, we decided to submit our sequence assigning it to a viral name. In previous work, the presence of ectopic DNA fragments derived from mitoviruses, infecting a fungal host (*Gigaspora margarita*), was detected but their function is still uncharacterised (Turina et al., 2018), contrary to analogous cDNA fragments found in insects and involved in anti-viral defense (Goic et al., 2016). Furthermore, RsMV22 is closely related to the viral dsRNA element isolated by Lakshman and co-authors in 1998, where a DNA stage was reported. To search for indications of mitovirus-derived DNA sequences in our samples, we analyzed DNAs extracted from the eight *R. solani* isolates by qPCR using the same primers used for the detection of the viruses (by qRT-PCR) after the bioinformatic analysis. No evidence of amplification was observed (data not shown). We designed primers for PCR amplification of around 300 base pair fragments on the genome of RsMV22, RsMV21, and RsMV24. Also, in this case, we were able to amplify and clone the fragments in *E. coli* using the cDNA template and confirming the sequence, while no amplification was observed using extracted DNA as template. A 300 bp fragment was amplified and also cloned for RsOLV5, demonstrating the presence of the viral sequence only in the cDNA and not in the genomic DNA. Overall, we could not provide evidence of the existence of DNA fragments corresponding to the *R. solani* mitovirus in the isolates we tested.

The four viruses named Rhizoctonia solani ourmia-like virus 2 to 5 were grouped together with the fungal ourmia-like viruses, with Agaricus bisporus virus 15 as the closest hit in a BLAST search (Figure 2). Putative RdRp produced by RsOLV 2 and RsOLV 3 showed a 77% identity between them, and therefore, they are likely different isolates of the same species. A recent proposal grouped fungal ourmiaviruses together with plant ourmiaviruses in a new family called *Botourmiaviridae*; inside this family, three different genera containing fungal ourmiaviruses are established: *Botoulivirus, Magoulivirus*, and *Scleroulivirus* (Figure 2). Our phylogenetic analysis shows that the new species identified in our study are part of a distinct clade containing also Agaricus bisporus virus 15, which appears to be basal to the proposed *Botourmiaviridae* family, therefore, posing the ground for a new virus family for which we propose the name Basidionarnaviridae, since currently it contains members infecting basidiomycetes.

### Hepe-Virga Group

**Figure 3:**
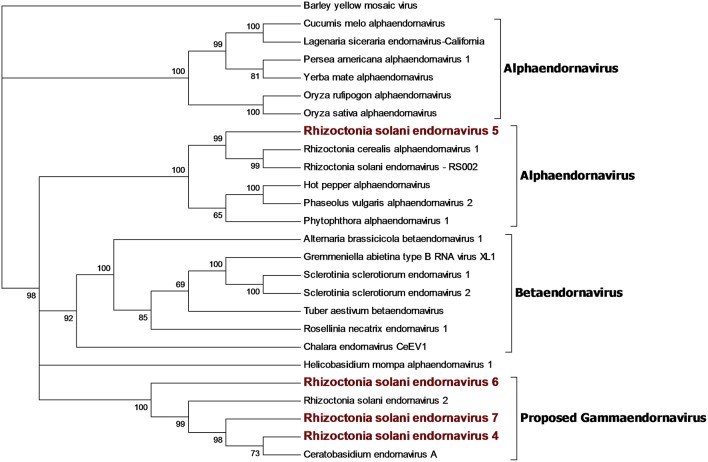
Phylogenetic analysis of viruses belonging to the family *Endornaviridae*. Twenty six viral sequences were used to build the alignment. A phylogenetic tree was built using the maximum likelihood method and the best substitution model selected by ModelFinder was VT+F+I+G4. Ultrafast bootstrap analysis was performed with 1,000 replicates and branches displaying values below 50 were collapsed. Viruses discovered in this work are outlined in bold red. Barley yellow mosaic virus, belonging to the family *Potyviridae*, was used as outgroup. A list of accession numbers of the sequences used for this analysis can be found in Supplementary Table 4, online. Detailed information about softwares used for the analysis can be found in the material and methods section.

Our bioinformatics pipeline unveiled four contigs encoding a single ORF showing similarity with viruses belonging to the *Endornaviridae* family. Phylogenetic analysis (Figure 3) on the predicted proteins shows that viral contigs are grouped together with viruses from the genus *Alphaendornavirus*, thus, we renamed the sequences as Rhizoctonia solani endornavirus 4 to 7. All the proteins predicted from the viral contigs show an RdRp domain, and proteins encoded from RsEV4, RsEV6, and RsEV7 also showed a helicase domain. A methyl-transferase domain was detected only in RsEV5 protein. In general, our endornavirus phylogenetic tree showed differences with the current taxonomic organization of this family, that we think requires an update to recognize new genera inside the family. *Endornavirus* RsEV4, RsEV6, and RsEV7 constitute a new clade that could possibly turn into a new genus, for which we propose the name Gammaendornavirus (Figure 3).

**Figure 4:**
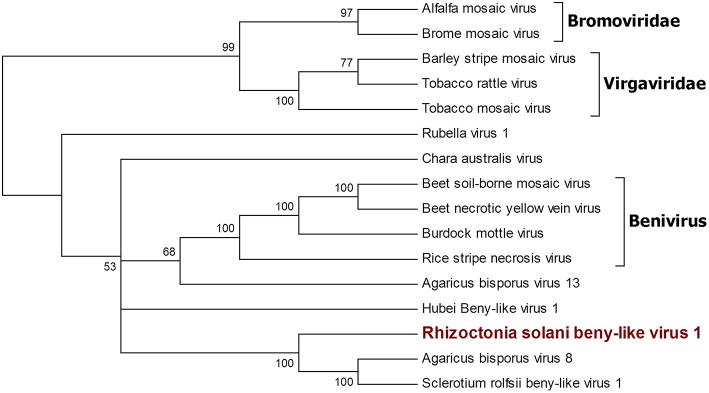
Phylogenetic analysis of viruses belonging to the family *Benyviridae*. Sixteen viral sequences were aligned and a phylogenetic tree was derived using the maximum likelihood method. The best substitution model selected by ModelFinder was VT+F+I+G4. Ultrafast bootstrap analysis was performed with 1,000 replicates, and branches displaying values below 50 were collapsed. Viruses discovered in this work are outlined in bold red. *Bromoviridae* and *Virgaviridae* were used as outgroups. A list of accession numbers of the sequences used for this analysis can be found in Supplementary Table 4, online. Detailed information about softwares used for the analysis can be found in the material and methods section.

A single 11.666-nt-long contig was identified as a new virus; an ORF prediction and BLAST analysis showed similarities with other characterized beny-like mycoviruses. Thus, the contig was named Rhizoctonia solani beny-like virus 1 (Figure 4). PCR with specific primers for RsBLV1 allowed us to amplify a 274 bp fragment from cDNA obtained from the infected isolates, while no specific amplification was observed on DNA extracted from the same isolate. Similarly to the still unpublished Sclerotium rolfsii beny-like virus 1, RsBLV1 encodes for one single ORF producing a 3,584-amino acid-long protein showing a viral helicase domain and an RdRp domain. Other beny-like mycoviruses like Agaricus bisporus virus 8 and 13 show three ORFs that were not detected in RsBLV1.

**Figure 5:**
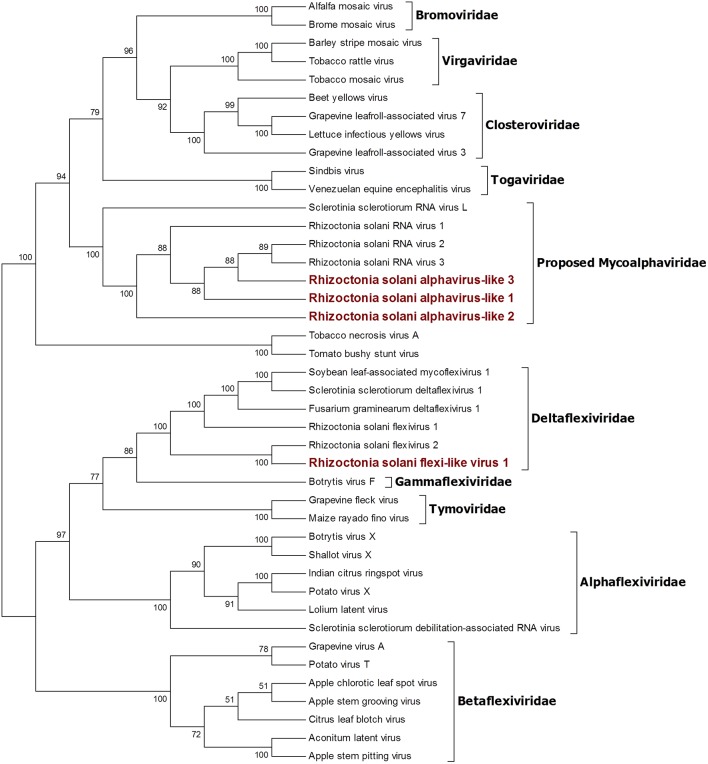
Phylogenetic analysis of viruses related to the Alphavirus supergroup. Forty two sequences were aligned and a phylogenetic tree was built using the maximum likelihood method. The best substitution model selected by ModelFinder was VT+F+I+G4 and ultrafast bootstrap analysis was performed with 1,000 replicates. Branches displaying values below 50 were collapsed. Viruses discovered in this work are outlined in bold red ink. A list of accession numbers of the sequences used for this analysis can be found in Supplementary Table 4, online. Detailed information about softwares used for the analysis can be found in the material and methods section.

Four contigs showed high similarity with viruses encoding ORFs with an RdRp domain belonging to the alphavirus supergroup (Wolf et al., 2018). Among these, a 2,982-nt-long contig encodes for an ORF displaying an RdRp domain and a viral helicase domain. BLAST and phylogenetic analysis show that the closest viruses to this contig is Rhizoctonia solani flexivirus 2, and these two viruses probably belong to the newly characterized genus *Deltaflexivirus* (Figure 5). Viruses belonging to the genus *Deltaflexivirus* are supposed to encode small proteins in the 3-terminal part of the genome region and are usually around 8 kbp-long; all these characteristics were not observed in our contig or in the Rhizoctonia solani flexivirus 2 (Bartholomäus et al., 2016), but we cannot exclude that these two genomes are indeed partial. Taken together these considerations, we decided to name this virus Rhizoctonia solani flexi-like virus 1. The three remaining contigs belonged to viruses that we named Rhizoctonia solani alphavirus-like 1 to 3; RsAVL1 and RsAVL2 encode for uncomplete ORFs encoding RdRp, while the protein predicted from RsAVL3 seems to be a complete RdRp. Phylogenetic analysis (Figure 5) showed that these viruses are grouped together with three very small partial viral genomes discovered in *Rhizoctonia solani* (Rhizoctonia solani RNA virus 1 to 3) that have high homology with our sequences (Bartholomäus et al., 2016). Together with Sclerotinia sclerotiorum RNA virus L, these viruses form a distinct clade for which we propose a new family called Mycoalphaviridae. PCR amplification of overlapping fragments of around 800 bp from RsALV 3 confirmed the absence of the viral contig in the DNA of the host fungal isolate, confirming the viral nature of this segment.

### Hypo Related Sequences

**Figure 6:**
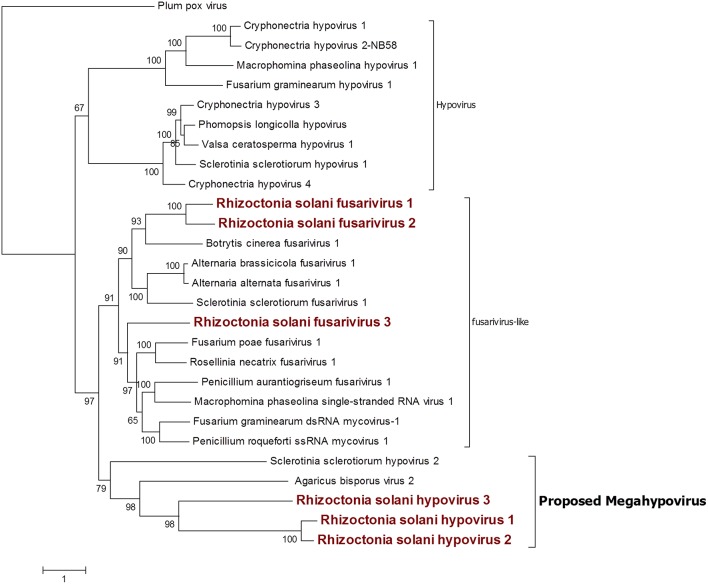
Phylogenetic analysis of viruses related to the family *Hypoviridae*. Twenty eight sequences were used to build an alignment and to derive a phylogenetic tree using the maximum likelihood method. The best substitution model selected by ModelFinder was VT+F+G4 and an ultrafast bootstrap analysis was performed with 1,000 replicates. Viruses discovered in this work are outlined in bold red. Plum pox virus, belonging to the family *Potyviridae*, was used as outgroup. A list of accession numbers of the sequences used for this analysis can be found in Supplementary Table 5, online. Detailed information about softwares used for the analysis can be found in the material and methods section.

Six contigs showed a relationship to the *Hypoviridae* family (Figure 6). Among those, three contigs were closely related to the genus *Hypovirus*, while the remaining three were more similar to the still unclassified viruses generally called fusarivirus in a number of publications; identity percentages resulting from BLASTx analysis showed levels between 28.5 and 40.5% compared to the first hit. No other clearly correlated Rhizoctonia solani hypoviruses and fusariviruses were found in the literature. Thus, we named these contigs Rhizoctonia solani hypovirus 1 to 3 and Rhizoctonia solani fusarivirus 1 to 3. RsHV1 had an 18,371-bp-long genome. The ORF prediction showed just one large putative protein of 5,344 amino acids where only one helicase domain can be detected. RdRp domains could not be observed and no GDD amino acid triplet, the hallmark of most viral RdRps, was found in the protein sequence. Nevertheless, from the BLAST analysis, it was clear that the RsHV1 protein had homology with the region encoding for the GDD domain in other hypoviruses like Sclerotinia sclerotiorum hypovirus 2 (for which an RdRp domain was annotated), even though such domain is not detected by common domain searching software, such as CDD sparkle and ExPASy-PROSITE (Sigrist et al., 2013; Marchler-Bauer et al., 2017). According to the International Committee on Taxonomy of Viruses—ICTV description, hypoviruses have a genome dimension of 9.1–12.7 kb. On the contrary, RsHV1 has one of the longest genomes known so far for a putative hypovirus. RsHV2 is a 9,606-bp contig encoding for two ORFs, but the 3′ proximal-ORF appears to be incomplete. Both ORFs did not show any conserved motif, although the protein amino acid sequence BLASTs with viruses belonging to the genus *Hypovirus*. RsHV3 is 5,518-bp-long with an ORF prediction which underlined two putative proteins of which the 3'proximal ORF was likely an incomplete protein displaying a viral helicase domain. No RdRp domain could be detected in this case, and no conserved domains were observed on the 5' distal ORF. Alignments performed to produce a phylogenetic tree showed that RsHV1 and RsHV3 could be aligned on the helicase domain, and that the phylogenetic tree resulting from the analysis show a clade containing RsHV1, RsHV2, RsHV3 related to a hypovirus from *Agaricus bisporus* (Agaricus bisporus virus 2) and Sclerotinia sclerotiorum hypovirus 2. These viruses are included in a statistically well supported clade that separates them from the current characterized fusariviruses and the members of the genus *Hypovirus* (Figure 6). For this reason, we propose here the name of a new genus (Megahypovirus) for the large size of the genomes of the two complete sequences so far characterized in this group (SsHV2 and RsHV1). The presence of RsHV3 was confirmed through RT-PCR amplification; we were able to amplify and clone a fragment of the expected size just in the cDNA produced from the infected fungal isolate, and nothing was observed in the DNA extracted from the same fungus—confirming its viral nature and that the transcript was not derived from an endogenised viral fragment.

Among the three fusariviruses discovered (Figure 6), RsFV1 was the longest one, as it had a 10,776-bp genome and the ORF prediction displayed four putative proteins. ORF 2 and 4 were the smallest: ORF 2 encoded a putative protein of 525 amino acids and ORF 4 encoded a putative protein of 578 amino acids. BLAST analysis and domain prediction could not find any convincing hit or conserved domain for both ORFs. ORF 1 was 731 amino acids long and it had a viral helicase domain, while the RdRp domain was found in ORF 3, together with another helicase domain. The same genome organization was observed for RsFV2, a 10,710-bp contig showing a PolyA site at the 3′ of the sequence and encoding for four putative proteins. In this virus, ORF 2 and 4 (472 and 718 amino acids, respectively) were short ORFs with no significant homology with other viral proteins. ORF1 had a viral helicase domain and ORF 3 had a helicase and an RdRp domain. Finally, RsFV3 was a 5959-bp contig encoding for just one protein. The predicted protein sequence showed helicase and RdRp domains as observed for the other two fusarivirus and a PolyA site at the 3′ end. As previously described, fusariviruses often encoded two ORFs, with the 5′-ORF encoding for the RdRp. The new viruses that we discovered in this work presented some different characteristics that are peculiar for this group of viruses (four ORFs in RsFV1 and RsFV2). We decided to further investigate the ORF prediction of RsFV1 and RsFV2 by designing PCR primers and amplifying sequences overlapping the regions of discontinuity between the four ORFs to verify possible mistakes in the sequence. Cloned fragments were sequenced and compared to the viral genomes proving that the reference sequence assembled from Trinity was identical to the one amplified through PCR. Thus, the four proteins predicted cannot be due to an error in the RNAseq assembly. Phylogenetic analysis confirmed BLAST analysis and placed our three fusariviruses together with others already characterized in the same clade. We propose that currently recognized fusariviruses, based on their sequence length and genome organization, be subdivided into at least two further genera (Figure 6).

### Bunya-Arena Like Sequences

**Figure 7:**
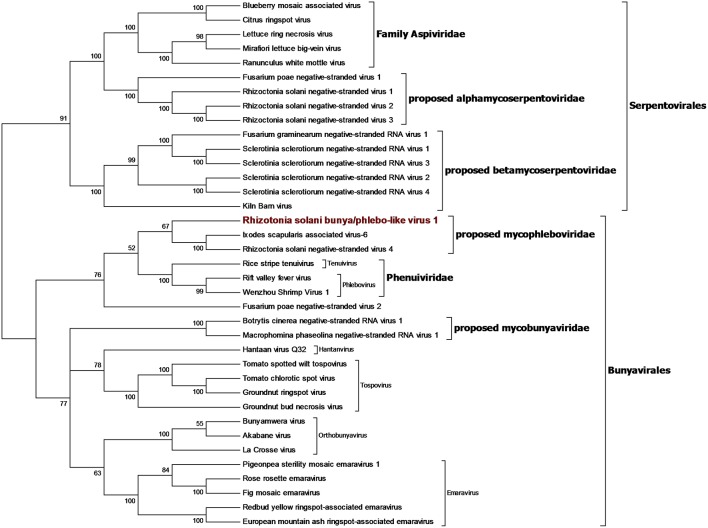
Phylogenetic analysis of viruses related to the orders *Bunyavirales* and *Serpentovirales*. Thirty seven sequences were aligned and the best substitution model selected by ModelFinder was VT+F+G4 and implemented in a maximum likelihood method to derive the Phylogenetic tree. Ultrafast bootstrap analysis was performed with 1,000 replicates and branches displaying values below 50 were collapsed. The Virus discovered in this work is outlined in red. A list of accession numbers of the sequences used for this analysis can be found in Supplementary Table 6, online. Detailed information about software used for the analysis can be found in the material and methods section.

We identified a contig encoding for a protein showing the RdRp domain from bunyaviruses and we named this contig Rhizoctonia solani bunya/phlebo-like virus 1. RsBPLV1 is 7,804 bp long and the putative RdRp is 2513 amino acids long. BLASTp analysis on the RdRp showed 30.59% identity with the closest virus in the database. Phylogenetic analysis previously carried out showed that the fungal negative single-strand RNA viruses belong to three orders: the order *Mononegavirales* and family *Mymonaviridae* (Liu et al., 2014), the order *Serpentovirales* which likely includes two families that we propose to name Alphamycoserpentoviridae and Betamycoserpentoviridae, and the order *Bunyavirales* that includes other mycoviruses. RsBPLV1 was clearly in this order, in our analysis (Figure 7). Nevertheless, two distinct new families that include mycoviruses can be proposed inside this order: for the one that includes RsBPLV1 we propose the name Mycophleboviridae, whereas for the other well supported clade, we propose the name Mycobunyaviridae.

### Partiti-Picobirna Sequence Group

**Figure 8:**
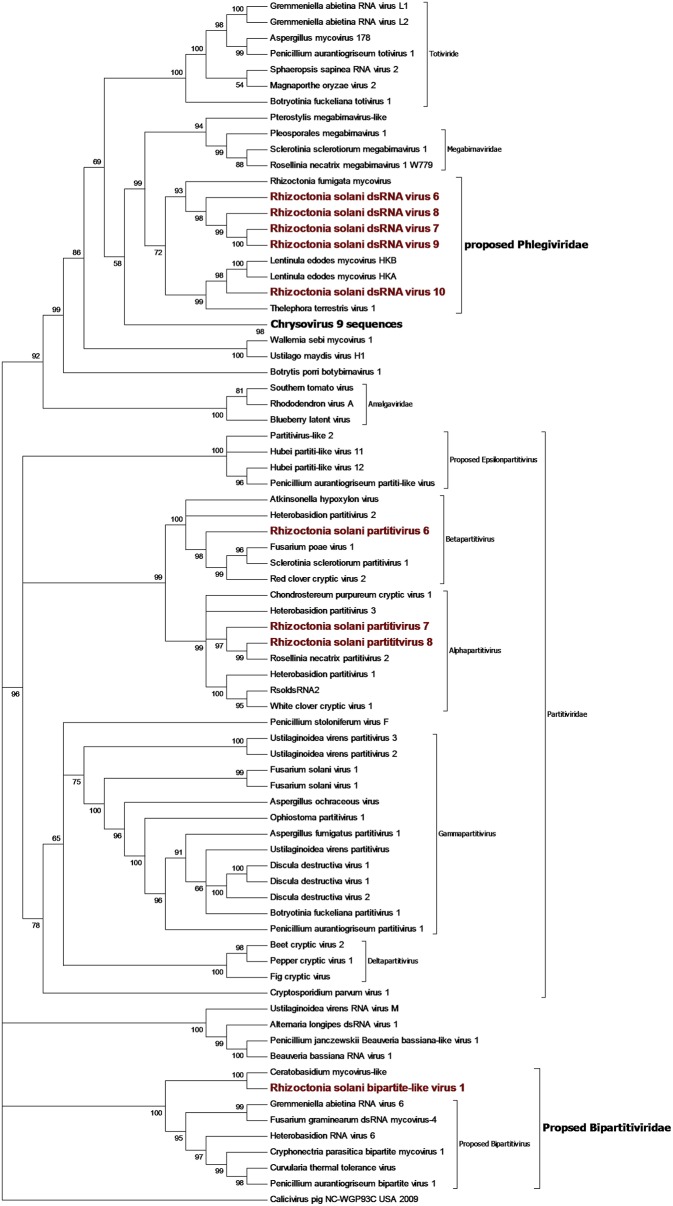
Phylogenetic analysis of dsRNA viruses. Eighty four sequences were used to build an alignment. Phylogenetic tree was built using the maximum likelihood method, the best substitution model selected by ModelFinder was Blosum62+F+G4 and ultrafast bootstrap analysis was performed with 1,000 replicates, branches displaying values below 50 were collapsed. Viruses discovered in this work are underlined in red. A list of accession numbers of the sequences used for this analysis can be found in Supplementary Table 7, online. Detailed information about software used for the analysis can be found in the material and methods section.

Contigs coding for proteins showing high homology with partitivirus RdRps were detected in our samples (Figure 8). From the initial BLAST analysis, Four contigs encoding for partitivirus RdRp and three contigs encoding for partitivirus coat proteins were retrieved. Among these seven contigs, one encoding for an RdRp and one encoding for a coat protein were easily matched as part of the same virus since they showed almost complete identity with an already characterized virus belonging to the genus *Alphapartitivirus* called Rhizoctonia solani dsRNA virus 2. Indeed, these contigs were always detected in the same fungal isolates through qRT-PCR, confirming that these two segments belonged to the same virus. The remaining contigs were submitted to the NCBI database as Rhizoctonia solani partitivirus 6 to 8. RsPV7 and RsPV8 RdRps were part of the genus *Alphapartitiviruses*, while RsPV6 belonged to the group hosting viruses from the genus *Betapartitivirus* (Figure 8). A correct correlation between RdRp and coat protein of RsPV6 and RsPV7 have been complicated by the fact that the two viruses were found in the same fungal isolate in our collection. Thus, we grouped together the two RNAs attributed to RsPV7 because the RdRp protein and the coat protein produced from the two segments had as a first BLAST hit the same two proteins from a single viral species called Trichoderma atroviride partitivirus 1 (Table 1). No RNA2 producing coat protein was detected initially for RsPV8, but we tried to look for the missing genome segment by a direct TBLASTn search on the RNAseq assembly using as a query the coat protein sequence from the virus whose RdRp is more similar to RsPV8 RdRp. We were able to find a short contig producing a protein that showed homology with partitivirus coat proteins, however, the coverage of this contig is low and attempts to extend the sequence with MITObim were not successful. Nevertheless, qRT-PCR analysis confirmed the presence of the contig only in the fungal isolate hosting the RsPV8 RNA1 fragment. Taken together, we submitted to Genbank the contig as RsPV8's partial RNA2.

Two short contigs of 1827 and 1888 bp were found as part of the same viral genome, where the 1827-bp contig encoded a single incomplete ORF producing a protein showing homology with RdRp from viruses with a bipartite genome related to a partitivirus that we have previously described (Nerva et al., 2016), and provisionally named bipartite viruses. The 1888-bp segment codes for a hypothetical protein, showing homology with the same virus group. Phylogenetic analysis (Figure 8) placed this virus basal to the virus group already proposed in literature as bipartite (Nerva et al., 2016), thus, we decided to rename this virus *R. solani* bipartite-like virus 1.

### Toti-Chryso

Five contigs encoded for putative RdRp with similarities to viruses belonging to the Toti-Chryso group (Figure 8). These contigs were renamed Rhizoctonia solani dsRNA virus 6 to 10, and phylogenetic analysis grouped these viruses together in a clade containing unclassified viruses related to the genus *Megabirnavirus*. This clade has already been proposed to form a new genus called Phlegivirus by previous work (Petrzik et al., 2016). Here, we show that such taxon comprises two genera, and therefore, we propose the family Phlegiviridae to include both of them (Figure 8). Rhizoctonia solani dsRNA 6 and 10 showed two ORFs; the first was a protein of unknown function, and the second ORF encoded for the RdRp. The RsdsRNA6 genome was 11,847 bp long: the first ORF was 2,300 amino acids long and the RdRp was a smaller protein of 1,125 amino acids. RsdsRNA 10 was 9,416 nt-long. The first ORF was 1,636 amino acids long, while the predicted RdRp was a 1,475-amino acid protein. No clear conclusion about genome organization of RsdsRNA 7 to 9 could be drawn since all three genomic segments encoded for a single ORF, the putative RdRps. Furthermore, RsdsRNA 9 ORF was partial, while for the other two ORFs, a stop codon could be detected. Since these three contigs were much shorter compared to the closest viruses according to the phylogenetic analysis, we can assume that they are probably incomplete. So far, attempts to extend the viral contigs were unsuccessful. PCR amplification of a target fragment from RsdsRNA10 gave positive results only when using the cDNA of the infected isolate as a template, while no PCR amplification was observed from total DNA, once again suggesting that the RNA was not derived from transcription of endogenised viral fragments.

### ORFans Fragments

Four fragments resulting from our bioinformatic pipeline were detected as viral but could not be located in any known taxonomical group, and no evidence of an RdRp domain was detected in these sequences. We called these contigs Rhizoctonia solani putative virus 1 to 4. RsPuV 1 was a 6,311-bp-long sequence, and the ORF encoded showed a conserved viral helicase domain, which has similarities with viruses from different groups like *Tymovirus* and *Endornaviruses*. Rhizoctonia solani putative virus 2 was 7,137 bp long, and in this case, the 2,083-amino acid long protein only had a conserved viral helicase motif. Rhizoctonia solani putative virus 3 had a 7,713-bp sequence, encoding for a 2,011-amino acid long uncomplete protein, showing a viral methyl transferase domain. BLAST analysis showed little homology with viruses from the genus *Tymovirus*. Rhizoctonia solani putative virus 4 had a 7,833-bp sequence coding for an ORF resulting in a putative 2,414-amino acid protein with a viral helicase domain. In each of these sequences, the homology with other viruses is always located on the small part of the protein showing the conserved domain (helicase and methyltransferase); thus, it is hard to hypothesize a specific taxonomic placement for these viral sequences. Indications that these fragments are of viral origin also comes from the fact that no DNA was detected corresponding to these fragments using DNA as template for PCR amplification (Supplementary Figure 2, online). We confirmed the presence of these putative virus RNA fragments through RT-PCR using cDNA as a template, which resulted in bands of the expected size. We cannot exclude that some of these viral fragments are part of a multipartite virus that has escaped our detection or are associated with one of the viruses we have described in this paper.

## Conclusions

In the present study we reported sequences corresponding to mostly new viral species belonging to the positive sense ssRNA genome virus groups (*Mitovirus, Botourmiaviridae, Hypovirus, Endornavirus*, Hepe-Virga-like), and to dsRNA virus groups such as the *Alphapartitivirus* and *Gammapartitivirus*. Furthermore, a single negative strand virus in the *Bunyavirales* was also characterized in our collection.

The number of viral species infecting each *R. solani* isolates ranged from 6 (IBRS 15) to 27 (IBRS23). The mitoviruses RsMV24 and RsMV29, and the endornavirus RsEV7, were the most prevalent viruses since they occurred in all the isolates. However, some mycoviruses were detected in only one isolate of *R. solani*: RsMV21, RsMV23, RsMV25, RsMV26, RsMV27, RsMV28, RsMV30, RsMV31, RsMV36, RsEV6, RsPV6, RsPV7, RsPV8, RsBPLV1, RsBLV1, RsBeLV1, RsALV3, RsOLV5, RsPuV4, RsFV1, RsHV2, and RsHV3.

Successful uses of mycoviruses have already been reported for the biological control of *C. parasitica* (Nuss, 1992) in natural conditions. Some promising hypovirulent strains were also detected for *Ophiostoma novo-ulmi* (Hong et al., 1999), *Fusarium graminearum* (Chu et al., 2002), *Botrytis cinerea* (Wu et al., 2007), and *Rosellinea necatrix* (Chiba et al., 2009).

The ability of fungi to cause disease in the host plant seems to not be affected by the wide diversity of the viral species detected in the same *Rhizoctonia* isolates, since all the isolates induced the same kind of symptoms in the *Z. japonica* host. Indeed, in greenhouse tests, the isolates IBRS11, IBRS19, and IBRS23 were the most pathogenic, while the isolate IBRS15 was the least pathogenic (Picarelli et al., in review). We could not correlate virus distribution to the geographical distribution of the isolates (Supplementary Figure 1, online), and it is surprising that the comprehensive characterization of the three viromes associated with *R. solani* so far have only a minimal overlap of identical sequences (Bartholomäus et al., 2016; Marzano et al., 2016). Nevertheless, some new taxonomical groups that include mostly *R. solani* viruses were common to the various studies as was the case of some subclades in the *Mitovirus* genus, the proposed Gammaendornavirus genus, the proposed Mycoalphaviridae family and the proposed Phlegiviridae family. Our study helps to define two completely new clades of mycoviruses, the proposed genus Megahypovirus and the family Basidiourmiaviridae.

## Data Availability

The datasets generated for this study can be found in GenBank, MK393902, MK393903, MK393904, MK393905, MK532273, MK532274, MK507781, MK507782, MK507783, MK507784, MK400668, MK400669, MK492913, MK492914, MK507788, MK507789, MK507790, MK507791, MK532272, MK507778, MK507779, MK507787, MK507793, MK507792, MK507786, MK372892, MK490928, MK375261, MK372893, MK372894, MK372895, MK372896, MK372897, MK372898, MK372899, MK372900, MK372901, MK372902, MK372903, MK490929, MK490930, MK372904, MK372905, MK372906, MK372907, MK372909, MK372908, MK507780, MK507785, MK532275, MK507793, MK558257, MK558256, MK558258, MK558259, MK558260, MK558255, and Bioproject PRJNA524447.

## Author Contributions

MP collected the samples, characterized the fungal isolates, checked their virulence, and extracted dsRNA. MF extracted the total RNA, carried out all the virus annotation, carried out RT-PCR and PCR, mapped reads on the genome, carried out the phylogenetic analysis, and wrote the manuscript. ER curated virus nomenclature and classification, and carried out some phylogenetic analyses and edited the manuscript. LN carried out the initial bionformatic virome characterization. MC extended the genome segments with MitoBIM. MT edited the manuscript and supervised LN, MC, and MF. AC supervised MP, planned the experiments and edited the manuscript and deposited sequences in the databases with ER.

### Conflict of Interest Statement

The authors declare that the research was conducted in the absence of any commercial or financial relationships that could be construed as a potential conflict of interest.
