# Supplementary material for: Extreme Diversity of Mycoviruses Present in Isolates of Rhizoctonia solani AG2-2 LP From Zoysia japonica From Brazil
Source: Front Cell Infect Microbiol. 2019 Jul 12;9:244. doi: 10.3389/fcimb.2019.00244 (PMC6640214; doi:10.3389/fcimb.2019.00244)
Supplement: Supplementary file 1 [file Data_Sheet_1.pdf]

Supplementary Table 1. List of primers used for quantitative RT-PCR (qRT-PCR) detection of viruses.

Virus Name		qRT-PCR primers			
Rhizoctonia solani endornavirus 4		For	5'-	CACTCCCCTGTCACTTGGAT	-3'
		Rev	5'-	ACCTAGTTAAACGCCGACCA	-3'
Rhizoctonia solani endornavirus 5		For	5'-	CATCTGCCAGACTTCCATGC	-3'
		Rev	5'-	CCACCGCACTATTCAGCTTC	-3'
Rhizoctonia solani endornavirus 6		For	5'-	TGTCTTACCTGCACCTCCAG	-3'
		Rev	5'-	GCTCATGAAGTTTGCCACCA	-3'
Rhizoctonia solani endornavirus 7		For	5'-	ACCAAGCTTCGAACTCAGGA	-3'
		Rev	5'-	ACGTGAAATTGGCTTGGCAT	-3'
Rhizoctonia solani partitivirus 8	RNA1	For	5'-	CATTTGGGTGCCGTGTTGTA	-3'
		Rev	5'-	CGACACTACCCTTGGGAACT	-3'
	RNA2	For	5'-	ATAGCCTCACGGAGAACCTG	-3'
		Rev	5'-	GTCCTATCTATGGTCCCGGC	-3'
Rhizoctonia solani partitivirus 6	RNA1	For	5'-	TCTTGTTGGGGAGGGGAATC	-3'
		Rev	5'-	CGAGTACTACTGCCGTCCAT	-3'
	RNA2	For	5'-	TTGTTCGTGTTGACAGCTCG	-3'
		Rev	5'-	AAACCACGTCAAACTGGCTC	-3'
Rhizoctonia solani partitivirus 7	RNA1	For	5'-	TCTGGCCACTACAAGCATCA	-3'
		Rev	5'-	GAGATTCATCCAACCGCCAC	-3'
	RNA2	For	5'-	TGATCTCTTGTCCTCGACCG	-3'
		Rev	5'-	AACGATTCCAGTTCATGCGG	-3'
Rhizoctonia solani dsRNA virus 2	RNA1	For	5'-	TTGATGCGTGCCACAACTAG	-3'
		Rev	5'-	CAACCCTACGCCTGAAATCG	-3'
	RNA2	For	5'-	TGTTGAGGAGTGGAGCGATT	-3'
		Rev	5'-	CCTGCGTCTAAAAGCTCGAC	-3'
Rhizoctonia solani bipartite-like virus 1	RNA1	For	5'-	GCCCGCATATAGAGTTCCCT	-3'
		Rev	5'-	TGACTTCCAACTTGCAAGCC	-3'
	RNA2	For	5'-	CGCCAGTACTCGAACCATTG	-3'
		Rev	5'-	CATCGGCGAAAACGGATTCT	-3'
Rhizoctonia solani dsRNA virus 6		For	5'-	CGGCGTGTAATGAGGAGTTG	-3'
		Rev	5'-	CGTAGAAAGTTCGAGCCAGC	-3'
Rhizoctonia solani dsRNA virus 7		For	5'-	ACTCATCCACCATCCCGTTT	-3'
		Rev	5'-	TGCCATCCCTGATTCCAAGT	-3'
Rhizoctonia solani dsRNA virus 8		For	5'-	ACCATCCTTGTCCCGAATGT	-3'
		Rev	5'-	CAGTACAATGTTCCGCCTCG	-3'
Rhizoctonia solani dsRNA virus 9		For	5'-	ACGCGGTTAACAAGCTTGAG	-3'
		Rev	5'-	GACTGATTCTCCACACCCCA	-3'
Rhizoctonia solani dsRNA virus 10		For	5'-	TCTAAGCCCGGCAAAGGTAA	-3'
		Rev	5'-	TCCTGACTACCCATCCCAGA	-3'
Rhizoctonia solani beny-like virus 1		For	5'-	TTCACTGAAGTTCTTGGTAAGCCTACTA	-3'
		Rev	5'-	TTTGAACGGCGATCTGGAA	-3'
Rhizoctonia solani bunya/phlebo-like virus 1		For	5'-	CCGCCACGAAGAGATACTTCA	-3'
		Rev	5'-	GGGCCCCGCCTTCA	-3'
Rhizoctonia solani flexi-like virus 1		For	5'-	CAATCACCAGCGTCTCGAAG	-3'
		Rev	5'-	CACTCAATGCTAACGAGGCC	-3'

Rhizoctonia solani alphavirus-like 1	For	5'-	TGAACCAGATGACCTCCTCG	-3'
	Rev	5'-	TCAATTTGACGTCTTGCCCG	-3'
Rhizoctonia solani alphavirus-like 2	For	5'-	AGTCGTCTCCAATCCCCATG	-3'
	Rev	5'-	CGATCTTCCTCAACACGACG	-3'
Rhizoctonia solani alphavirus-like 3	For	5'-	TGGAGAAGTGTGTTGCGTTG	-3'
	Rev	5'-	GTAGGGGTGGTTCAGTGTCA	-3'
Rhizoctonia solani mitovirus 21	For	5'-	CCTTCCGGTGTTAGACGAGA	-3'
	Rev	5'-	AACCGAGCACAAACTGGAAC	-3'
Rhizoctonia solani mitovirus 22	For	5'-	TGTATGCACAGCCAGAGGAA	-3'
	Rev	5'-	AGTGTGATGAGTCCGCTTCA	-3'
Rhizoctonia solani mitovirus 23	For	5'-	GGAGACGTACCGAAAGCTCT	-3'
	Rev	5'-	TCCGTCAAAGAGCTCCATCA	-3'
Rhizoctonia solani mitovirus 24	For	5'-	AGGTCCACAATCGGGTCTTT	-3'
	Rev	5'-	GGATAGCCCCATTGAGTCGA	-3'
Rhizoctonia solani mitovirus 25	For	5'-	TTGGTTCAAGGCAAAGTCCG	-3'
	Rev	5'-	AGGTGAGGGCTTTGTGATGA	-3'
Rhizoctonia solani mitovirus 26	For	5'-	GCTTGACCGTTGATGGCTAC	-3'
	Rev	5'-	TCTATGAAGGTCGACGCAGG	-3'
Rhizoctonia solani mitovirus 27	For	5'-	GTTGGGCATTTGTTCCGTCT	-3'
	Rev	5'-	AAGGCAAGAGTCGTGGGTAT	-3'
Rhizoctonia solani mitovirus 28	For	5'-	TGCCCCTTGGTTAGCATTTG	-3'
	Rev	5'-	GCACAATGAAAGAAGGGGCA	-3'
Rhizoctonia solani mitovirus 29	For	5'-	TAGCTCCCATTGGTTGTCCC	-3'
	Rev	5'-	GGGATTTGGTGAAGCTTGGA	-3'
Rhizoctonia solani mitovirus 30	For	5'-	TGCCCCTTGGTTAGCATTTG	-3'
	Rev	5'-	GCACAATGAAAGAAGGGGCA	-3'
Rhizoctonia solani mitovirus 31	For	5'-	AATAAGGTCCTCGGCTCACC	-3'
	Rev	5'-	GACTAACCCTTCCCAGCAGT	-3'
Rhizoctonia solani mitovirus 32	For	5'-	TTACAGAACTCCATGGCCGT	-3'
	Rev	5'-	GCGGTCGCGAAAAGATACTT	-3'
Rhizoctonia solani mitovirus 33	For	5'-	AATAGTGCCGCCCAGTGATA	-3'
	Rev	5'-	TCTTGACCTTACTGCAGCGA	-3'
Rhizoctonia solani mitovirus 34	For	5'-	GTTGTCCAACGGCGTATCTC	-3'
	Rev	5'-	ATACGCGGCACCTTTTGAAA	-3'
Rhizoctonia solani mitovirus 35	For	5'-	CCCCGCAGACTCTCTTACAA	-3'
	Rev	5'-	TCTGCTAGTGACCCGGTTTT	-3'
Rhizoctonia solani mitovirus 36	For	5'-	TAGAGGCGGCTTCATCATCC	-3'
	Rev	5'-	GATTCAACAGGTTTCGGGCA	-3'
Rhizoctonia solani mitovirus 37	For	5'-	GTTAGTTCATGGCTCGCTGG	-3'
	Rev	5'-	AATCATCACCCAGGACAGCA	-3'
Rhizoctonia solani mitovirus 38	For	5'-	GGGTGGAAAGTTGCAGGAAG	-3'
	Rev	5'-	GTGTAAGACTCAGCGCCAAC	-3'
Rhizoctonia solani ourmia-like virus 2	For	5'-	AGGCAGTTCGAAGTCCTCAA	-3'
	Rev	5'-	GTCTCACCTCATCGCCGATA	-3'
Rhizoctonia solani ourmia-like virus 3	For	5'-	CAATTGTAGTCCTGTCGCCG	-3'
	Rev	5'-	GTGATATCGAGAGGGACCGG	-3'
Rhizoctonia solani ourmia-like virus 4	For	5'-	CAATCATGTCTGCGGGGAAG	-3'

	Rev	5'-	GAGCATGAGTCCATTGAGCG	-3'
Rhizoctonia solani ourmia-like virus 5	For	5'-	ACCGAAGTCCCGTTCACTAATATC	-3'
	Rev	5'-	GGGTGATGGCCCGTGTTAT	-3'
Rhizoctonia solani fusarivirus 1	For	5'-	ATGTACGCCAAGGTCAATGC	-3'
	Rev	5'-	TGATCATTGGCAGCATCGTG	-3'
Rhizoctonia solani fusarivirus 2	For	5'-	TTTCTTGACAACGCTCCTGC	-3'
	Rev	5'-	ACCCAGAATTGATGCCAGGA	-3'
Rhizoctonia solani fusarivirus 3	For	5'-	CCGCATACCTTGGTCGAAAG	-3'
	Rev	5'-	CCTGTTAATGTGCGGACGAG	-3'
Rhizoctonia solani hypovirus 1	For	5'-	TTGACACGCTTCACATGCAA	-3'
	Rev	5'-	GCGTTCGAGTTGGAATCCTC	-3'
Rhizoctonia solani hypovirus 2	For	5'-	GCGAATTACATGGCCGAGTT	-3'
	Rev	5'-	AAGATGTCGTGCAATACCGC	-3'
Rhizoctonia solani hypovirus 3	For	5'-	AACCTTGGCCGGCTTGAC	-3'
	Rev	5'-	TGGATAAGGAAGATGTGGCAGAT	-3'
Rhizoctonia solani putative virus 1	For	5'-	CATGCCATTGAATGCTGTAACA	-3'
	Rev	5'-	CCAGTGACTGCAGCATTTTCTG	-3'
Rhizoctonia solani putative virus 2	For	5'-	GATACGTGAGCACGGCTATGTC	-3'
	Rev	5'-	CGTCATCGTTCCTACTGCTAAGC	-3'
Rhizoctonia solani putative virus 3	For	5'-	GCCGCCTTCCAGTTGGT	-3'
	Rev	5'-	GGGACTTCCAGTGGCACAA	-3'
Rhizoctonia solani putative virus 4	For	5'-	CAATGGTTGGGCTGAAACG	-3'
	Rev	5'-	CGCACACAGGCCAACTCAT	-3'

Supplementary Table 2. List of primers used for PCR amplification of fragments from the viral genomes in the fungal cDNA and genomic DNA. For each primer set, the fragment length is reported in the size column.

Primer set name	size (bp)	Primers			
Rhizoctonia solani putative virus 1	248	For	5'-	TGTCCATTTTCCCCGCAAAG	-3'
		Rev	5'-	CCCAGTGACTGCAGCATTTT	-3'
Rhizoctonia solani putative virus 2	228	For	5'-	CGTCCTTATGCAACTTCCGG	-3'
		Rev	5'-	ACTCAGAGGGCGATTTGACA	-3'
Rhizoctonia solani putative virus 3	256	For	5'-	AGTCCTCGAAGCTGTCAACA	-3'
		Rev	5'-	ATCAACCGCATTAAGACGCC	-3'
Rhizoctonia solani putative virus 4	251	For	5'-	GCAGCTATTCTATCGGCGTG	-3'
		Rev	5'-	CTACGATTGCAATGTCCGGG	-3'
Rhizoctonia solani dsRNA virus 10	379	For	5'-	CAGCTTGGCATCTGGGATTC	-3'
		Rev	5'-	CCACATCGCTAGCCCTCATA	-3'
Rhizoctonia solani bunya/phlebo-like virus 1	254	For	5'-	CCTGATGGTGAAGAAGGGGT	-3'
		Rev	5'-	TCTCCTGCTTCTTGTCCTGG	-3'
Rhizoctonia solani mitovirus 21	291	For	5'-	GGGCACGCAGAGACAGTATA	-3'
		Rev	5'-	AGAGGGGTGGTTGAATGAGG	-3'
Rhizoctonia solani mitovirus 22	278	For	5'-	GGGCCTATTCCATGAGCTCT	-3'
		Rev	5'-	GCTTCGCCGTCTAACAAAGT	-3'
Rhizoctonia solani mitovirus 23	244	For	5'-	ACTCGCTATCATACAGGAGGC	-3'
		Rev	5'-	GGCAACCGGTAATCTGTCTG	-3'
Rhizoctonia solani ourmia-like virus 5	254	For	5'-	TCGAAAAGGTCCTCTGCCAT	-3'
		Rev	5'-	GTCGACCTGAAATTCCGCTC	-3'
Rhizoctonia solani beny-like virus 1	274	For	5'-	CAATCTCCATCACCGCCAAG	-3'
		Rev	5'-	TTGAAACCTTGCACGACAGG	-3'
Rhizoctonia solani hypovirus 3	222	For	5'-	CGATTGGGGCTTCACTTACG	-3'
		Rev	5'-	GCCTGAATTCTGACGCTGAG	-3'
Rhizoctonia solani alphavirus-like 3 set 1	867	For	5'-	TCTACGAGGGTGGCGTAGAA	-3'
		Rev	5'-	ACTTGCCATCATCTGGGGTG	-3'
Rhizoctonia solani alphavirus-like 3 set 2	780	For	5'-	CGTGGACTCACCAATCCCAA	-3'
		Rev	5'-	CGTCGTCGCTGACATCCTTA	-3'
Rhizoctonia solani alphavirus-like 3 set 3	729	For	5'-	AGACGAGGGAGTACCAGACC	-3'
		Rev	5'-	AGAGCTACTTCGCCGAAACC	-3'
Rhizoctonia solani alphavirus-like 3 set 4	861	For	5'-	GTTTCGGAACGCACCACAAA	-3'
		Rev	5'-	GTTCACCGTTATTGAGCGGC	-3'
Rhizoctonia solani fusarivirus 1 junction 1	418	For	5'-	AATGACCCACTGAGTGCTGG	-3'
		Rev	5'-	TTTTGACGGCGGATGGAGAA	-3'
Rhizoctonia solani fusarivirus 1 junction 2	518	For	5'-	TGCTGCCAATGATCATCGTG	-3'
		Rev	5'-	TGACGGCTTCCTCAATGTATG	-3'
Rhizoctonia solani fusarivirus 1 junction 3	473	For	5'-	TCGAGGGACTGCTTGTCCTA	-3'
		Rev	5'-	CTGGCCCACCTTTCAGATGT	-3'
Rhizoctonia solani fusarivirus 2 junction 1	416	For	5'-	TTGCACAGGTCGGTGTTGAT	-3'
		Rev	5'-	ACCACAGACAACAAAGGCCA	-3'
Rhizoctonia solani fusarivirus 2 junction 2	565	For	5'-	CACCTGACGTTTGAACAAGC	-3'
		Rev	5'-	GATGGTTCCTGGTAAACGGC	-3'
Rhizoctonia solani fusarivirus 2 junction 3	468	For	5'-	GGCAAAGGCACTTGTTCTGG	-3'

		Rev	5'-	TAGCCACTCCATACCCGACA	-3’
Rsolani β tubulin	362	For	5'-	TAGGCCAGAGAAAGCGGAAA	-3’
		Rev	5'-	AACTCGGCCTACTTTGTCGA	-3’

Supplementary Table 3. List of accession used for alignment and phylogenetic inference of Narna-levi related viruses.

Accession	Virus name
AQM32767.1	Agaricus bisporus mitovirus 1
AQM49945.1	Agaricus bisporus virus 15
DAB41740.1	Ambrosia artemisiifolia mitovirus 1
DAB41741.1	Azolla filiculoides mitovirus 1
BPR53122.1	Alternaria arborescens mitovirus 1
AKN79252.1	Alternaria brassicicola mitovirus
ASM94071.1	Barns Ness breadcrumb sponge narna-like virus 6
ASM94072.1	Barns Ness breadcrumb sponge narna-like virus 7
ASM94073.1	Barns Ness breadcrumb sponge narna-like virus 8
YP 009333144.1	Beihai narna-like virus 3
YP 009333266.1	Beihai narna-like virus 4
YP 009333251.1	Beihai narna-like virus 6
APG77081.1	Beihai narna-like virus 7
YP 009333278.1	Beihai narna-like virus 11
AVH76945.1	Beta vulgaris mitovirus 1
CEZ26297.1	Botrytis cinerea mitovirus 2
CEZ26298.1	Botrytis cinerea mitovirus 3
CEZ26310.1	Botrytis ourmia-like virus
AHY03257.1	Buergenerula spartinae mitovirus 1
DAB41756.2	Cannabis sativa mitovirus 1
ACI03053.1	Cassava virus C
APG77188.1	Changjiang narna-like virus 3
AWL21855.1	Chenopodium quinoa mitovirus 1
CCG47524.1	Clitocybe odora virus
AMQ67414.1	Cronartium ribicola mitovirus 1
AMQ67415.1	Cronartium ribicola mitovirus 2
AMQ67416.1	Cronartium ribicola mitovirus 3
AMQ67417.1	Cronartium ribicola mitovirus 4
AMQ67418.1	Cronartium ribicola mitovirus 5
AAR01971.1	Cryphonectria cubensis mitovirus 1b
AAA61703.1	Cryphonectria parasitica mitovirus 1-NB631
DAB41747.1	Dahlia pinnata mitovirus 1
AAD17381.1	dsRNA viral RNA-dependent RNA polymerase (mitochondrion) Thanatephorus cucumeris
CAA27499.1	Enterobacteria phage GA
CAA30375.1	Enterobacteria phage SP
ACF16357.1	Epirus cherry virus
DAB41748.1	Erigeron breviscapus mitovirus 1
NP 040650.1	Escherichia virus MS2
NP 046752.1	Escherichia virus Qbeta
AHI43533.1	Fusarium circinatum mitovirus 1
AHI43534.1	Fusarium circinatum mitovirus 2-1
BAV56289.1	Fusarium poae mitovirus 1
BAV56290.1	Fusarium poae mitovirus 2
BAV56291.1	Fusarium poae mitovirus 3
BAV56292.1	Fusarium poae mitovirus 4
AVA17449.1	Gigaspora margarita mitovirus 1
AVA17450.1	Gigaspora margarita mitovirus 2

AVA17451.1	Gigaspora margarita mitovirus 3
AVA17452.1	Gigaspora margarita mitovirus 4
AAT48883.1	Gremmeniella abietina mitocondrial RNA virus S2
AEY76153.1	Gremmeniella abietina non-host-specific mitochondrial RNA virus S1
BAD72871.1	Helicobasidium mompa mitovirus 1-18
AIF33766.2	Heterobasidion mitovirus 1
YP 009336796.1	Hubei narna-like virus 2
YP 009337787.1	Hubei narna-like virus 3
YP 009336672.1	Hubei narna-like virus 5
APG77208.1	Hubei narna-like virus 10
YP 009336759.1	Hubei narna-like virus 12
DAB41749.1	Humulus lupulus mitovirus 1
AIU44705.1	Hymenoscyphus fraxineus mitovirus 1
ALD89100.1	Macrophomina phaseolina mitovirus 1
AMM45292.1	Macrophomina phaseolina mitovirus 3
SBQ28480.1	Magnaporthe oryzae ourmia-like virus
CAJ32466.1	Ophiostoma mitovirus 1a
CAJ32467.1	Ophiostoma mitovirus 1b
CAA06228.1	Ophiostoma mitovirus 3a
CAJ32468.1	Ophiostoma mitovirus 3b
CAB42652.1	Ophiostoma mitovirus 4
CAB42653.1	Ophiostoma mitovirus 5
CAB42654.1	Ophiostoma mitovirus 6
AGT55877.1	Ophiostoma mitovirus 7
ACF16360.1	Ourmia melon virus
DAB41745.1	Oxybasis rubra mitovirus 1
DAB41744.1	Petunia exserta mitovirus 1
YP 009345044.1	Phomopsis longicolla RNA virus 1
YP 009241365.1	Phytophthora infestans RNA virus 4
AIT71973.1	Rhizoctonia cerealis mitovirus
AHL25281.1	Rhizoctonia mitovirus 1 RS002
ALD60243.1	Rhizoctonia mitovirus K1
ANA08076.1	Rhizoctonia oryzae-sativae mitovirus 1
ALD89121.1	Rhizoctonia solani mitovirus 2
ALD89125.1	Rhizoctonia solani mitovirus 6
ALD89127.1	Rhizoctonia solani mitovirus 8
ALD89116.1	Rhizoctonia solani mitovirus 11
ALD89117.1	Rhizoctonia solani mitovirus 12
ALD89118.1	Rhizoctonia solani mitovirus 13
ALD89120.1	Rhizoctonia solani mitovirus 15
MK372892	Rhizoctonia solani mitovirus 21
MK490928	Rhizoctonia solani mitovirus 22
MK375261	Rhizoctonia solani mitovirus 23
MK372893	Rhizoctonia solani mitovirus 24
MK372894	Rhizoctonia solani mitovirus 25
MK372895	Rhizoctonia solani mitovirus 26
MK372896	Rhizoctonia solani mitovirus 27
MK372897	Rhizoctonia solani mitovirus 28
MK372898	Rhizoctonia solani mitovirus 29
MK372899	Rhizoctonia solani mitovirus 30
MK372900	Rhizoctonia solani mitovirus 31
MK372901	Rhizoctonia solani mitovirus 32
MK372902	Rhizoctonia solani mitovirus 33
MK372903	Rhizoctonia solani mitovirus 34
MK490929	Rhizoctonia solani mitovirus 35
MK490930	Rhizoctonia solani mitovirus 36
MK372904	Rhizoctonia solani mitovirus 37
MK372905	Rhizoctonia solani mitovirus 38

ALD89131.1	Rhizoctonia solani ourmia-like virus 1 RNA 1
MK372906	Rhizoctonia solani ourmia-like virus 2
MK372907	Rhizoctonia solani ourmia-like virus 3
MK372908	Rhizoctonia solani ourmia-like virus 4
MK372909	Rhizoctonia solani ourmia-like virus 5
BAN85985.1	Rhizophagus sp. HR1 mitovirus-like ssRNA
BAJ23143.2	Rhizophagus sp. mitovirus RF1
AAC98708.1	Saccharomyces 23S RNA narnavirus
AHX72146.1	Sclerotinia sclerotiorum mitovirus 1 HC025
AGC24231.1	Sclerotinia sclerotiorum mitovirus 2
AGC24232.1	Sclerotinia sclerotiorum mitovirus 3
AMT92141.1	Sclerotinia sclerotiorum mitovirus 4
AHF48622.1	Sclerotinia sclerotiorum mitovirus 6
AHX84135.1	Sclerotinia sclerotiorum mitovirus 7
AHF48624.1	Sclerotinia sclerotiorum mitovirus 8
AHF48625.1	Sclerotinia sclerotiorum mitovirus 9
AHF48627.1	Sclerotinia sclerotiorum mitovirus 11
AHF48628.1	Sclerotinia sclerotiorum mitovirus 12
AHF48631.1	Sclerotinia sclerotiorum mitovirus 15
ALD89138.1	Sclerotinia sclerotiorum ourmia-like virus 1 RNA 1
ALD89139.1	Sclerotinia sclerotiorum ourmia-like virus 2 RNA 1
YP 009336532.1	Shahe narna-like virus 3
DAB41743.1	Solanum chacoense mitovirus 1
ALM62238.1	Soybean leaf-associated ourmiavirus 1
ALM62250.1	Soybean leaf-associated ourmiavirus 2
AAT09164.1	Thielaviopsis basicola mitovirus
AEG79311.1	Tuber aestivum mitovirus
AEP83726.1	Tuber excavatum mitovirus
YP 009337193.1	Wenling narna-like virus 2
APG77283.1	Wenzhou narna-like virus 1
YP 009336518.1	Wenzhou narna-like virus 2
YP 009336520.1	Wenzhou narna-like virus 3
YP 009333318.1	Wenzhou narna-like virus 4
YP 009337619.1	Wenzhou narna-like virus 9
APG76981.1	Wenzhou narna-like virus 11
APG77163.1	Wenzhou shrimp virus 10
YP 009344979.1	Wuhan spider virus 7

Supplementary Table 4. List of accession used for alignment and phylogenetic inference of positive single-stranded RNA viruses from Hepe-Virga group (*Endornaviridae*, *Benyviridae*, Alphavirus supergroup).

Accession	Virus name
Endornaviridae	
YP 009115493.1	Alternaria brassicicola betaendornavirus 1
NP 148999.1	Barley yellow mosaic virus
YP 009310113.1	Ceratobasidium endornavirus A
vADN43901.1	Chalara endornavirus CeEV1
YP 009222598.1	Cucumis melo alphaendornavirus
YP 529670.1	Gremmeniella abietina type B RNA virus XL1
YP 003280846.1	Helicobasidium mompa alphaendornavirus 1
YP 009165596.1	Hot pepper alphaendornavirus
YP 009010973.1	Lagenaria siceraria endornavirus-California
YP 438202.1	Oryza rufipogon alphaendornavirus
YP 438200.1	Oryza sativa alphaendornavirus
YP 005086952.1	Persea americana alphaendornavirus 1
ALJ56098.1	Phaseolus vulgaris alphaendornavirus 2
YP 241110.1	Phytophthora alphaendornavirus 1
YP 008719905.1	Rhizoctonia cerealis alphaendornavirus 1
AHL25280.1	Rhizoctonia solani endornavirus - RS002
AMM45288.1	Rhizoctonia solani endornavirus 2
MK393902	Rhizoctonia solani endornavirus 4
MK393903	Rhizoctonia solani endornavirus 5
MK393904	Rhizoctonia solani endornavirus 6
MK393905	Rhizoctonia solani endornavirus 7
BAT32944.1	Rosellinia necatrix endornavirus 1
YP 008169851.1	Sclerotinia sclerotiorum endornavirus 1
AND83000.1	Sclerotinia sclerotiorum endornavirus 2
YP 004123950.1	Tuber aestivum betaendornavirus
YP 009046830.1	Yerba mate alphaendornavirus
Benyviridae	
AQM49930.1	Agaricus bisporus virus 8
AQM49942.1	Agaricus bisporus virus 13
YP 053235.1	Alfalfa mosaic virus
NP 604481.1	Barley stripe mosaic virus
NP 612615.1	Beet necrotic yellow vein virus
YP 009513207.1	Beet soil-borne mosaic virus
NP 041197.1	Brome mosaic virus
YP 008219063.1	Burdock mottle virus
AEJ33768.1	Chara australis virus
APG77690.1	Hubei Beny-like virus 1
MK507778	Rhizoctonia solani beny-like virus 1
ABU94739.2	Rice stripe necrosis virus
NP_062883.2	Rubella virus 1
AZF86092.1	Sclerotium rolfsii beny-like virus 1
AIL54434.1	Tobacco mosaic virus
AHG52750.1	Tobacco rattle virus
***Alphavirus* supergroup**	
NP 116487.1	Aconitum latent virus
YP 053235.1	Alfalfa mosaic virus
ABY71563.1	Apple chlorotic leaf spot virus
BAA98054.1	Apple stem grooving virus
AEP02955.1	Apple stem pitting virus
NP 604481.1	Barley stripe mosaic virus
NP733949.1	Beet yellows virus
NP 068549.1	Botrytis virus F
NP 932306.1	Botrytis virus X
NP 041197.1	Brome mosaic virus

NP 624333.1	Citrus leaf blotch virus
YP 009268710.1	Fusarium graminearum deltaflexivirus 1
NP 542612.1	Grapevine fleck virus
NP 813795.3	Grapevine leafroll-associated virus 3
YP 004935919.1	Grapevine leafroll-associated virus 7
AAO17778.1	Grapevine virus A
NP 203553.1	Indian citrus ringspot virus
Q83045.2	Lettuce infectious yellows virus
YP 001718499.1	Lolium latent virus
NP 115454.1	Maize rayado fino virus
AAF89747.1	Potato virus X
BAM16482.1	Potato virus T
ANR02702.1	Rhizoctonia solani RNA virus 1
ANR02701.1	Rhizoctonia solani RNA virus 2
ANR02700.1	Rhizoctonia solani RNA virus 3
MK507793	Rhizoctonia solani alphavirus-like virus 1
MK507792	Rhizoctonia solani alphavirus-like 2
MK507786	Rhizoctonia solani alphavirus-like 3
MK507787	Rhizoctonia solani flexi-like virus 1
ANR02698.1	Rhizoctonia solani flexivirus 1
ANR02703.1	Rhizoctonia solani flexivirus 2
ACE88957.1	Sclerotinia sclerotiorum RNA virus L
YP 325662.1	Sclerotinia sclerotiorum debilitation-associated RNA virus
YP 009508363.1	Sclerotinia sclerotiorum deltaflexivirus 1
NP 620648.1	Shallot virus X
AGT56188.1	Sindbis virus
YP 009508374.1	Soybean leaf-associated mycoflexivirus 1
AIL54434.1	Tobacco mosaic virus
ADE10194.1	Tobacco necrosis virus A
AHG52750.1	Tobacco rattle virus

Supplementary Table 5. List of accession used for alignment and phylogenetic inference of *Hypovirus* and *Fusarivirus*-like.

Accession	Virus name
AQM49946.1	Agaricus bisporus virus 2
BBD71147.1	Alternaria alternata fusarivirus 1
YP 009222009.1	Alternaria brassicicola fusarivirus 1
YP 009480678.1	Botrytis cinerea fusarivirus 1
NP 041091.1	Cryphonectria hypovirus 1
AAA20137.1	Cryphonectria hypovirus 2-NB58
NP 051710.1	Cryphonectria hypovirus 3
YP 138519.1	Cryphonectria hypovirus 4
AAT07067.2	Fusarium graminearum dsRNA mycovirus-1
YP 009011065.1	Fusarium graminearum hypovirus 1
YP 009272906.1	Fusarium poae fusarivirus 1
ALD89099.1	Macrophomina phaseolina hypovirus 1
ALD89094.1	Macrophomina phaseolina single-stranded RNA virus 1
YP 009182154.1	Penicillium aurantiogriseum fusarivirus 1
YP 009052456.1	Penicillium roqueforti ssRNA mycovirus 1
YP 009051683.1	Phomopsis longicolla hypovirus
NP 040807.1	Plum pox virus
MK558257	Rhizoctonia solani fusarivirus 1
MK558256	Rhizoctonia solani fusarivirus 2
MK558258	Rhizoctonia solani fusarivirus 3
MK558259	Rhizoctonia solani hypovirus 1
MK558260	Rhizoctonia solani hypovirus 2
MK558255	Rhizoctonia solani hypovirus 3
YP 009047147.1	Rosellinia necatrix fusarivirus 1
YP 009143301.1	Sclerotinia sclerotiorum fusarivirus 1
YP 004782527.1	Sclerotinia sclerotiorum hypovirus 1
AIA61616.1	Sclerotinia sclerotiorum hypovirus 2
YP 005476604.1	Valsa ceratosperma hypovirus 1

Supplementary Table 6. List of accession used for alignment and phylogenetic inference of Bunya-Arenavirus group.

Accession	Virus name
BAF57206.1	Akabane virus
AIF28241.1	Blueberry mosaic associated virus
CEZ26311.1	Botrytis cinerea negative-stranded RNA virus 1
AKX73309.1	Bunyamwera virus
Q6DN67.1	Citrus ringspot virus
YP_003104764.1	European mountain ash ringspot-associated emaravirus
AEI98676.1	Fig mosaic emaravirus
ATP75709.1	Fusarium graminearum negative-stranded RNA virus 1
YP_009272911.1	Fusarium poae negative-stranded virus 1
YP_009272912.1	Fusarium poae negative-stranded virus 2
ATP79732.1	Groundnut bud necrosis virus
AMZ00269.1	Groundnut ringspot virus
ABD28179.1	Hantaan virus Q32
AUW34408.1	Ixodes scapularis associated virus-6
AWA82236.1	Kiln Barn virus
NP_671968.1	La Crosse Virus
AAT09109.1	Lettuce ring necrosis virus
ALD89106.2	Macrophomina phaseolina negative-stranded RNA virus 1
AAN60447.1	Mirafiori lettuce big-vein virus
CCP46989.1	Pigeonpea sterility mosaic emaravirus 1
AAK69629.2	Ranunculus white mottle virus
AEO95760.1	Redbud yellow ringspot-associated emaravirus
MK507779	Rhizoctonia solani bunya/phlebo-like V 1
ALD89129.2	Rhizoctonia solani negative-stranded virus 1
ALD89130.1	Rhizoctonia solani negative-stranded virus 2
ALD89111.1	Rhizoctonia solani negative-stranded virus 3
ALD89133.1	Rhizoctonia solani negative-stranded virus 4
ALD89133.1	Rhizoctonia solani negative-stranded virus 4
BAA06677.1	Rice stripe tenuivirus
P27316.1	Rift valley fever virus
YP 004327589.1	Rose rosette emaravirus
YP 009094317.1	Sclerotinia sclerotiorum negative-stranded RNA virus 1
ALD89145.1	Sclerotinia sclerotiorum negative-stranded RNA virus 2
AJT39503.1	Sclerotinia sclerotiorum negative-stranded RNA virus 3
ALD89140.1	Sclerotinia sclerotiorum negative-stranded RNA virus 4
YP009408637.1	Tomato chlorotic spot virus
BAD86755.1	Tomato spotted wilt virus
YP_009304989.1	Wenzhou Shrimp Virus 1

Supplementary Table 7. List of accession used for alignment and phylogenetic inference of dsRNA viruses (*Partitivirus-Picobirnavirus* group and *Totivirus-Chrysovirus* group).

Accession	Virus name
YP 009052469.1	Alternaria longipes dsRNA virus 1
CAH03664.1	Amasya cherry disease associated chrysovirus
CAY25801.2	Aspergillus fumigatus partitivirus 1
ABX79995.1	Aspergillus mycovirus 178
ABX79996.1	Aspergillus mycovirus 1816
ABV30675.1	Aspergillus ochraceous virus
AAA61829.1	Atkinsonella hypoxylon virus
YP 009154711.1	Beauveria bassiana RNA virus 1
ADP24757.1	Beet cryptic virus 2
YP 003934623.1	Blueberry latent virus
YP 009353026.1	Botryosphaeria dothidea chrysovirus 1
YP 001686789.1	Botryotinia fuckeliana partitivirus 1
YP 001109580.1	Botryotinia fuckeliana totivirus 1
YP 006390636.1	Botrytis porri botybirnavirus 1
ADG27878.1	Calicivirus pig NC-WGP93C USA 2009
AOX47586.1	Ceratobasidium mycovirus-like
CAQ53729.1	Chondrostereum purpureum cryptic virus 1
ACT79255.1	Cryphonectria nitschkei chrysovirus 1
AGK89731.1	Cryphonectria parasitica bipartite mycovirus 1
AAC47805.1	Cryptosporidium parvum virus 1
YP 001976143.1	Curvularia thermal tolerance virus
AAG59816.1	Discula destructiva virus 1
NP 116716.1	Discula destructiva virus 1
NP 620301.1	Discula destructiva virus 2
CBW77436.1	Fig cryptic virus
YP 003288790.1	Fusarium graminearum dsRNA mycovirus-4
ABQ53134.1	Fusarium oxysporum chrysovirus 1
AAC98734.1	Fusarium poae virus 1
BAA09520.1	Fusarium solani virus 1
NP 624350.1	Fusarium solani virus 1
NP 624332.2	Gremmeniella abietina RNA virus L1
YP 044807.1	Gremmeniella abietina RNA virus L2
AIU98624.1	Gremmeniella abietina RNA virus 6
ADV15441.1	Heterobasidion partitivirus 1
ADL66905.1	Heterobasidion partitivirus 2
ACO37245.1	Heterobasidion partitivirus 3
AEX87903.2	Heterobasidion RNA virus 6
YP 009329875.1	Hubei partiti-like virus 11
APG78257.1	Hubei partiti-like virus 12
BAM34028.1	Lentinula edodes mycovirus HKA
BAJ21197.1	Lentinula edodes mycovirus HKB
YP 003858286.1	Magnaporthe oryzae chrysovirus 1
YP 001649206.1	Magnaporthe oryzae virus 2
CAJ31886.1	Ophiostoma partitivirus 1
AOR51389.1	Partitivirus-like 2
YP 009182335.1	Penicillium aurantiogriseum bipartite virus 1
YP 009182157.1	Penicillium aurantiogriseum partiti-like virus
YP 009182336.1	Penicillium aurantiogriseum partitivirus 1
YP 009212848.1	Penicillium aurantiogriseum totivirus 1
ALO50135.1	Penicillium janczewskii Beauveria bassiana-like virus 1
YP 009182332.1	Penicillium janczewskii chrysovirus 1
ALO50149.1	Penicillium janczewskii chrysovirus 2
AAU95758.1	Penicillium stoloniferum virus F
AEJ07890.1	Pepper cryptic virus 1

ALO50147.1	Pleosporales megabirnavirus 1
AOX47597.1	Pterostylis megabirnavirus-like
AGJ83765.1	Red clover cryptic virus 2
AJE29745.1	Rhizoctonia fumigata mycovirus
MK492913	Rhizoctonia solani bipartite-like virus 1
MK507788	Rhizoctonia solani dsRNA virus 6
MK507789	Rhizoctonia solani dsRNA virus 7
MK507790	Rhizoctonia solani dsRNA virus 8
MK507791	Rhizoctonia solani dsRNA virus 9
MK532272	Rhizoctonia solani dsRNA virus 10
MK507781	Rhizoctonia solani partitivirus 6
MK507783	Rhizoctonia solani partitivirus 7
MK532273	Rhizoctonia solani partitivirus 8
MK400668	Rhizoctonia solani dsRNA virus 2
YP 003868436.1	Rhododendron virus A
YP 003288763.1	Rosellinia necatrix megabirnavirus 1 W779
AB569997	Rosellinia necatrix partitivirus 2
YP 009143529.1	Sclerotinia sclerotiorum megabirnavirus 1
AFR78160.1	Sclerotinia sclerotiorum partitivirus 1
YP 002321509.1	Southern tomato virus
NP 047560.1	Sphaeropsis sapinea RNA virus 2
YP 009209482.1	Thelephora terrestris virus 1
AGO04402.1	Ustilaginoidea virens partitivirus
YP 008327312.1	Ustilaginoidea virens partitivirus 2
AGJ03719.1	Ustilaginoidea virens partitivirus 3
YP 009094186.1	Ustilaginoidea virens RNA virus M
NP 620728.1	Ustilago maydis virus H1
ADG21213.1	Verticillium dahliae chrysovirus 1
ALO50138.1	Wallemia sebi mycovirus 1
AAU14888.1	White clover cryptic virus 1

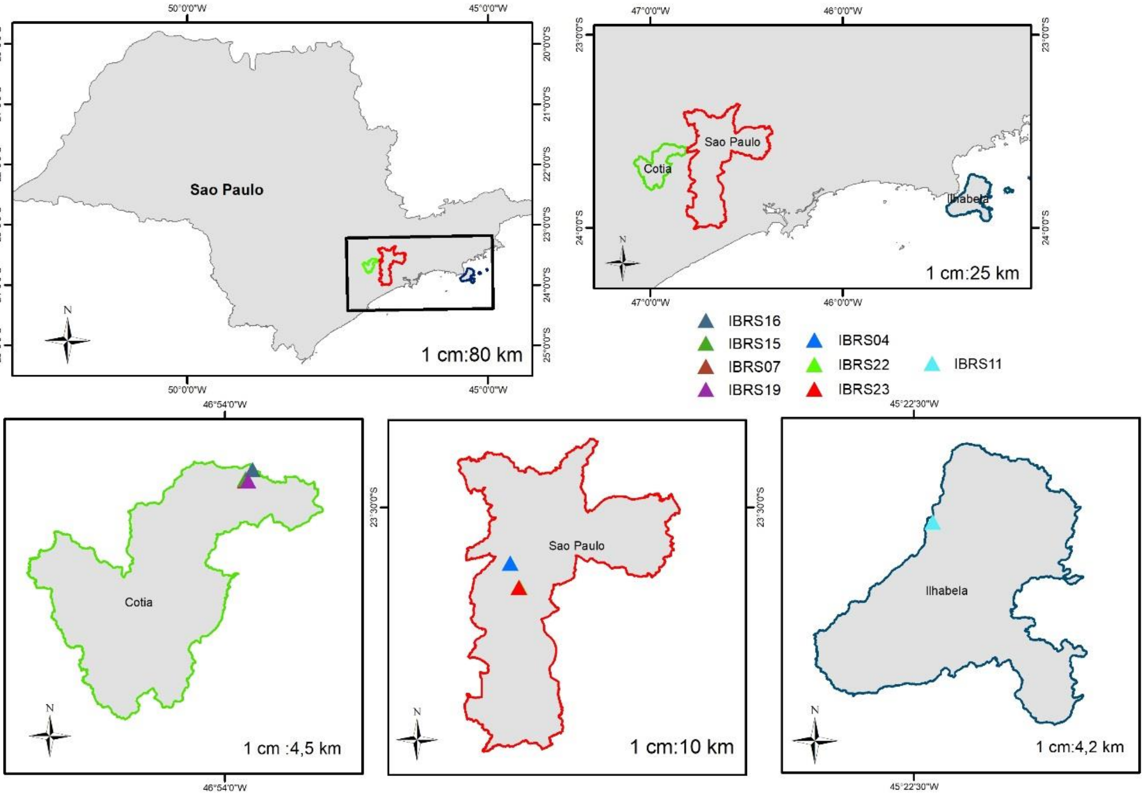

Supplementary on line Figure 1. Maps of the Sao Paulo State (Brazil) with the counties where the different Rhizoctonia solani isolates have been collected.

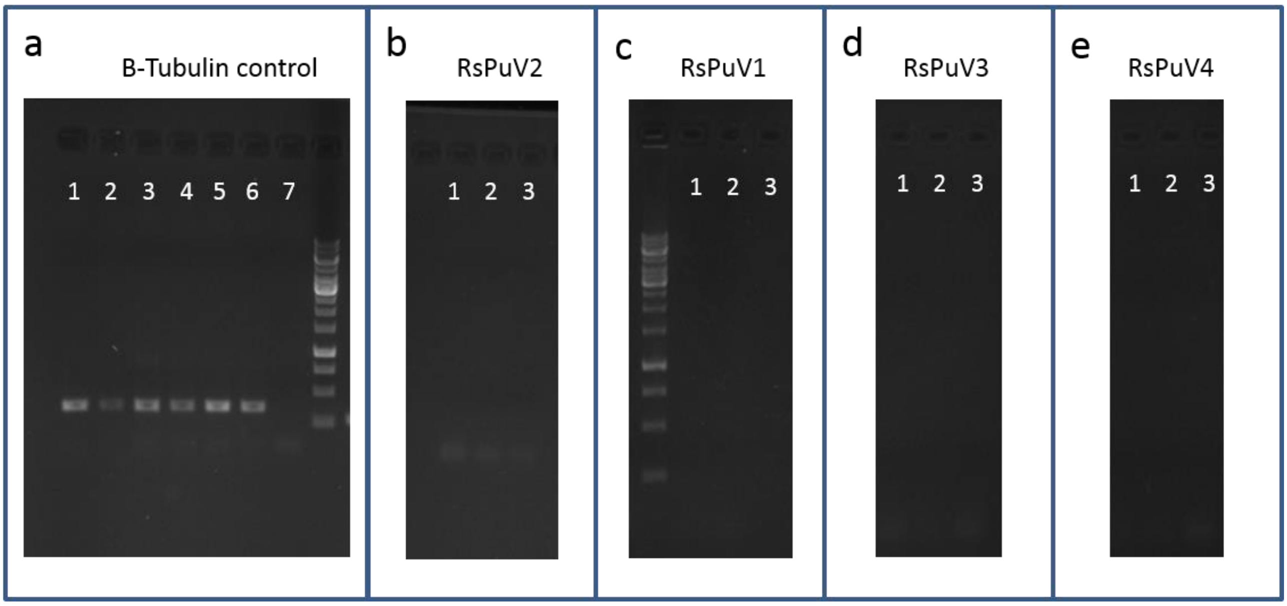

Supplementary on line Figure 2. Picture showing the electrophoresis gel obtained using primers for the four putative viruses (Supplementary on line Table 2) and the fungal DNA as template. In each of the reactions, a control reaction on the DNA extracted from uninfected isolate was performed to observe any clear unspecific amplification and exclude any endogenization event. **Panel a**: genomic control gene for the extracted DNA. DNAs extracted from 6 of the 8 isolates studied was tested for PCR reaction using primers for *R. solani* β tubulin gene. 1: IBRS23, 2: IBRS07, 3: IBRS11, 4: IBRS15, 5: IBRS16, 6: IBRS19, 7: No template control. **Panel b**: PCR amplification using the primer set for RsPV2, electrophoresis run is the same as for panel a; only primer dimers can be detected in the picture. 1: DNA from IBRS19, 2: DNA from IBRS11, 3: No template control. **Panel c**: PCR amplification using the primer set for RsPV1; only primer dimers can be detected in the picture; 1: DNA from IBRS07, 2: DNA from IBRS11, 3: No template control. **Panel d**: PCR amplification using the primer set for RsPV3, electrophoresis run is the same as for panel c; only primer dimers can be detected in the picture; 1: DNA from IBRS07, 2: DNA from IBRS11, 3: No template control. **Panel e**: PCR amplification using the primer set for RsPV4, electrophoresis run is the same as for panel c; only primer dimers can be detected in the picture. 1: DNA from IBRS07, 2: DNA from IBRS11, 3: No template control
